# RicePilaf: a post-GWAS/QTL dashboard to integrate pangenomic, coexpression, regulatory, epigenomic, ontology, pathway, and text-mining information to provide functional insights into rice QTLs and GWAS loci

**DOI:** 10.1093/gigascience/giae013

**Published:** 2024-06-04

**Authors:** Anish M S Shrestha, Mark Edward M Gonzales, Phoebe Clare L Ong, Pierre Larmande, Hyun-Sook Lee, Ji-Ung Jeung, Ajay Kohli, Dmytro Chebotarov, Ramil P Mauleon, Jae-Sung Lee, Kenneth L McNally

**Affiliations:** Bioinformatics Lab, Advanced Research Institute for Informatics, Computing and Networking, College of Computer Studies, De La Salle University, Manila 1004, Philippines; International Rice Research Institute (IRRI), Metro Manila 1301, Philippines; Bioinformatics Lab, Advanced Research Institute for Informatics, Computing and Networking, College of Computer Studies, De La Salle University, Manila 1004, Philippines; Bioinformatics Lab, Advanced Research Institute for Informatics, Computing and Networking, College of Computer Studies, De La Salle University, Manila 1004, Philippines; DIADE, Univ Montpellier, Cirad, IRD, 34394 Montpellier, France; National Institute of Crop Science, Wanju-gun 55365, Republic of Korea; National Institute of Crop Science, Wanju-gun 55365, Republic of Korea; International Rice Research Institute (IRRI), Metro Manila 1301, Philippines; International Rice Research Institute (IRRI), Metro Manila 1301, Philippines; International Rice Research Institute (IRRI), Metro Manila 1301, Philippines; International Rice Research Institute (IRRI), Metro Manila 1301, Philippines; International Rice Research Institute (IRRI), Metro Manila 1301, Philippines

**Keywords:** rice, GWAS, QTL analysis, post-GWAS, coexpression network, transcription factor binding, text mining

## Abstract

**Background:**

As the number of genome-wide association study (GWAS) and quantitative trait locus (QTL) mappings in rice continues to grow, so does the already long list of genomic loci associated with important agronomic traits. Typically, loci implicated by GWAS/QTL analysis contain tens to hundreds to thousands of single-nucleotide polmorphisms (SNPs)/genes, not all of which are causal and many of which are in noncoding regions. Unraveling the biological mechanisms that tie the GWAS regions and QTLs to the trait of interest is challenging, especially since it requires collating functional genomics information about the loci from multiple, disparate data sources.

**Results:**

We present RicePilaf, a web app for post-GWAS/QTL analysis, that performs a slew of novel bioinformatics analyses to cross-reference GWAS results and QTL mappings with a host of publicly available rice databases. In particular, it integrates (i) pangenomic information from high-quality genome builds of multiple rice varieties, (ii) coexpression information from genome-scale coexpression networks, (iii) ontology and pathway information, (iv) regulatory information from rice transcription factor databases, (v) epigenomic information from multiple high-throughput epigenetic experiments, and (vi) text-mining information extracted from scientific abstracts linking genes and traits. We demonstrate the utility of RicePilaf by applying it to analyze GWAS peaks of preharvest sprouting and genes underlying yield-under-drought QTLs.

**Conclusions:**

RicePilaf enables rice scientists and breeders to shed functional light on their GWAS regions and QTLs, and it provides them with a means to prioritize SNPs/genes for further experiments. The source code, a Docker image, and a demo version of RicePilaf are publicly available at https://github.com/bioinfodlsu/rice-pilaf.

Key Points

- Quantitative trait locus (QTL) analysis and genome-wide association studies (GWASs) in rice have identified a large number of loci–trait associations.
- Making sense of GWAS/QTL mapping results is challenging due to the large number of genes being implicated and the complex patterns of interactions among genes to produce a trait.
- RicePilaf cross-references GWAS/QTL-mapping results with multiple data sources on rice to provide insights into GWAS regions and QTLs.
- RicePilaf is free, open-source, and containerized. It can be run locally on a browser or can be set up as a web service.

## Background

Rice is a global food staple feeding half of humanity. To address the dual concerns of meeting the demands of a growing world population while minimizing contribution to climate change, scientists and breeders are continually seeking genetic sources for high-yield, sustainable, and robust rice varieties [1]. Paramount to this task are the identification and elucidation of the genetic and molecular basis of agronomically important traits.

Genome-wide association studies (GWASs) and quantitative trait locus (QTL) analysis in rice have identified a multitude of genetic loci influencing a wide range of important traits. Recent examples include GWAS/QTL analysis on variations in environmental stress responses (e.g., cold tolerance [2], heat tolerance [3, 4], and anaerobic germination [5, 6]), disease tolerance (e.g., blast [7]), mineral contents (e.g., cadmium accumulation [8]), morphological traits (e.g., plant height [9], grain weight [10], and grain size and panicle length [11]), and other yield-related traits (e.g., preharvest sprouting [12] and seed longevity [13]). This list is only expected to grow given the immense interest in the genetics of rice traits and the easy availability of genotyping resources covering millions of single-nucleotide polymorphisms (SNPs) across thousands of rice varieties [14].

The biological interpretation of statistical loci–trait associations is challenging due to a number of reasons. First, a typical GWAS can implicate hundreds to thousands of SNPs due to high linkage disequilibrium (LD)—estimates of LD extend to hundreds of kilobases for some rice populations [15, 16]. QTL mappings and GWAS peaks likewise may have tens to hundreds of underlying genes. Not all of the SNPs/genes in the reported loci will be causal, requiring a mechanism to narrow down the candidate list. Next, complex traits are likely influenced by multiple SNPs/genes, which individually are only able to explain a small amount of variation. Teasing out biological meaning therefore requires taking into account coexpression and coregulation patterns of groups of genes.

Furthermore, there might be many associated SNPs that are in intergenic and noncoding regions, given that a majority of SNPs in genotyping assays and genotype databases do not lie inside gene models [17, 18]. This requires incorporating regulatory information in the post-GWAS analysis. Lastly, since SNP genotypes are typically called against the Nipponbare reference, GWAS/QTLs are typically reported only in Nipponbare coordinates, even though several high-quality genome assemblies of a variety of accessions are now available. Given that a large number of between-population genomic variations have been reported [14], a pangenomic view of gene sets implicated by GWAS/QTL mapping is necessary.

Thus, the post-GWAS/QTL mapping task of prioritizing genes or identifying biological mechanisms that link them to the phenotype requires the integration of GWAS/QTL mapping results with genomic information from a host of other data sources. While tools for computational post-GWAS analysis have been reported for other species (e.g., [19, 20]), there are a limited number of tools dedicated to rice, one of which is Rice Galaxy [21] (now folded into CropGalaxy [22]), which utilizes genome position information and lift-over across a few rice genomes.

**Figure 1:**
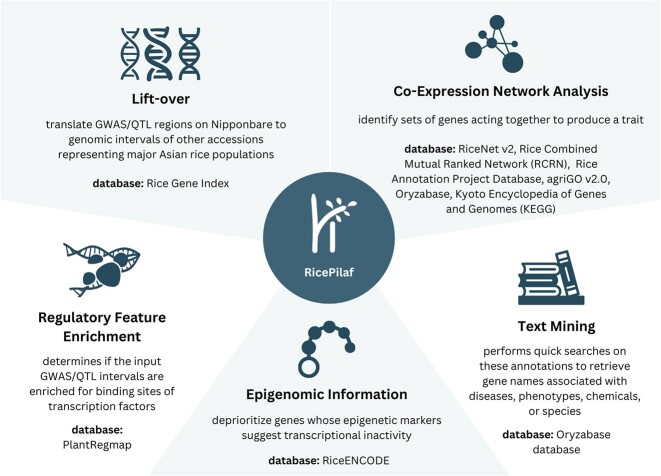
RicePilaf graphical abstract. RicePilaf crosses GWAS/QTL-mapping results with multiple data sources on rice. A summary of databases used in RicePilaf is provided in Table 4.

Here we report RicePilaf, a web app for post-GWAS/QTL analysis that integrates rice GWAS/QTL mapping results with pangenomic, coexpression, epigenomic, ontology, pathway, regulatory, and literature-mining information coming from various data sources and produces interactive web reports allowing users to, for example, search and sort data tables, move and click network nodes to display information on pertinent genes, and download the analysis results in text format (Fig. 1). It is built using the Python-based Dash and Flask frameworks. All dependencies are bundled into a Docker image; hence, it works on any of the major operating systems. It can be run locally on a web browser or provided as a web service. It is free and open source. Further details of the software are provided in the Availability of Source Code and Requirements section. We demonstrate the utility of the software using recent GWAS/QTL analysis on 2 key traits connected to modern rice cultivation: yield under drought and preharvest sprouting.

## Results and Discussion

### RicePilaf overview

**Figure 2:**
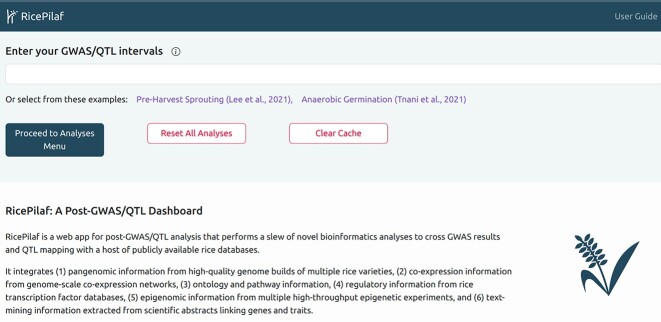
A screenshot of the input interface.

RicePilaf takes in as input a set of genomic intervals (Fig. 2), obtained from a QTL analysis or from clumping of LD-linked statistically significant SNPs from a GWAS, for example, computed by the LD-clumping procedure of PLINK [23]. It performs a series of novel bioinformatics analyses on the input intervals, which we overview here and describe in more detail in the Methods section.

#### Gene list

RicePilaf begins by retrieving the gene models overlapping the input intervals in the Nipponbare reference. For each gene model, it provides the following: (i) gene description and orthology information obtained from the Rice Gene Index (RGI) [24]; (ii) protein, protein domains, and protein family information from UniProt [25], InterPro [26], and Pfam [27], obtained by automated queries using PyRice [28]; and (iii) scientific literature associating the gene to traits, obtained from QTARO [29] and our in-house text-mined dataset.

#### Lift-over

Nipponbare serves as the gold-standard reference genome sequence and genomic coordinate system. Given that genotype calls are made against the Nipponbare reference, GWAS/QTL mapping results are reported in Nipponbare coordinates. Recently, high-quality genome builds of several accessions have been published [30–32]. Comparative genomics has revealed an abundance of duplications, deletions, insertions, inversions, and translocations during the evolution of rice [14, 33, 34].

For GWAS/QTL analysis on populations that are not derived from or include Nipponbare, by relying only on its genome and its annotation, we likely will miss genes and regulatory features linked to the phenotype of interest. Examples of genes of agronomic importance that are absent in Nipponbare (but present in indica or aus) include *Sub1* [35].

**Figure 3:**
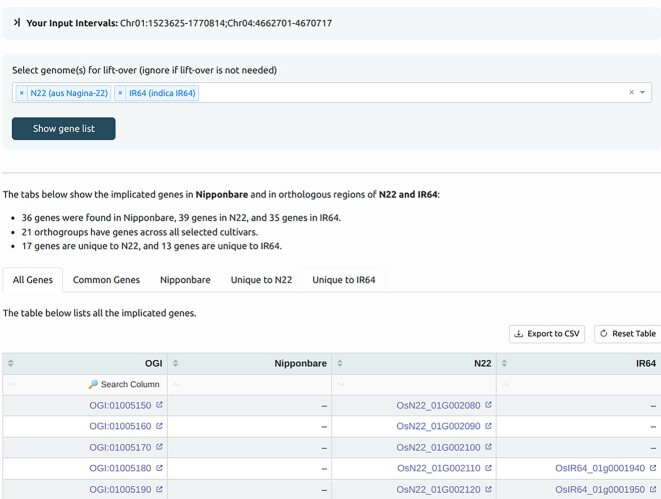
A screenshot showing an example of the result of lift-over from Nipponbare coordinates to another genome, IR64 in this case.

RicePilaf can lift over the intervals in the Nipponbare reference coordinates to several other recently published genomes, representing major rice populations. Using the RGI [24] database, it retrieves the genes overlapping the lifted-over intervals and their orthologs. This pangenomic view of gene sets may be useful if, for example, the GWAS/QTL mapping is on an accession that is closer to a genome other than Nipponbare (Fig. 3).

#### Coexpression network analysis

Complex traits are influenced by hundreds of SNPs/genes that individually are only able to explain a small amount of variation in the trait. Genes with the same or similar biological functions or involved in the same pathway are likely to be also coexpressed [36–38]. Coexpression networks provide a means to identify sets of genes acting together to produce a trait. An additional benefit of a coexpression network is that for genes with poor annotations or unknown functions, their membership in a dense subnetwork containing well-characterized genes might be a way to uncover incomplete functional information. Furthermore, coexpression networks have been used for post-GWAS analysis in a number of plants and animals [20, 39, 40].

**Figure 4:**
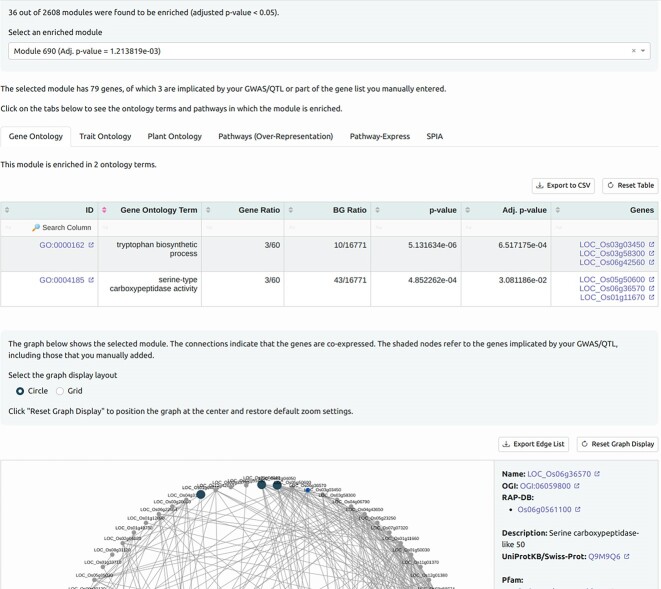
A screenshot showing an example of coexpression network analysis in RicePilaf.

To identify genes that may be acting collectively to result in a trait, RicePilaf searches rice coexpression networks, RiceNet v2 [41] and RCRN [42], for modules (communities or clusters) of genes that are statistically enriched in the genes overlapping the input intervals. Functional characterization of the modules is done via enrichment analysis against several ontology and pathway databases from agriGO [43], KEGG [44], and Oryzabase [45] (Fig. 4).

#### Regulatory feature enrichment

A majority of rice SNPs in genotyping assays and genotype databases are located in noncoding regions [14, 17, 18]. For example, the 3,000 Rice Genomes Project found twice as many SNPs in intergenic regions than within genes [46]. Unsurprisingly, GWAS/QTL mappings also report many noncoding trait-associated variants. It is likely that these influence the activity of regulatory elements. One possible causal link is that variants could alter transcription factor binding affinity, leading to changes in the expression of target genes, ultimately resulting in phenotypic variation  [47]. Post-GWAS tools for human data that overlap GWAS results with regulatory features such as transcription factor (TF) binding sites, chromatin accessibility, and histone marks [48, 49] have been previously reported.

To investigate variants that might be affecting the binding activity of transcription factors, RicePilaf searches for transcription factors whose known/predicted binding sites provided by PlantRegMap [50] significantly overlap with the input intervals.

#### Text mining

**Figure 5:**
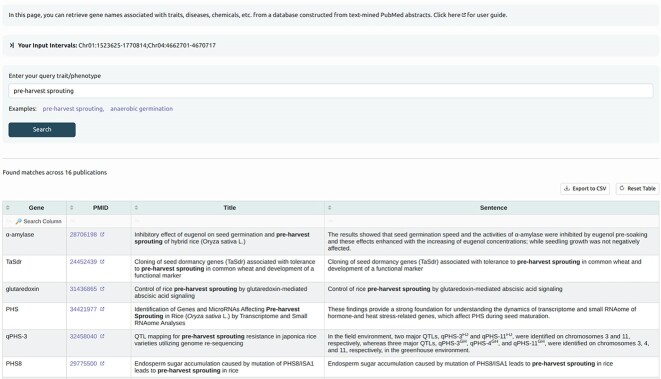
A screenshot showing an example of the result of searching against a text-mining derived database containing information from PubMed abstracts.

The gene list provided for the coexpression and regulatory enrichment analyses can be supplemented by genes retrieved from the pangenome lift-over and from querying our in-house dataset obtained by text mining PubMed abstracts on rice gene–trait associations (Fig. 5). Additionally, the same text-mining dataset is used to find scientific literature related to the genes overlapping the input interval.

#### Epigenomic information

For traits that are tissue specific, it may be desirable to deprioritize genes whose epigenetic markers suggest transcriptional inactivity. Using the embeddable Integrative Genomics Viewer [51], RicePilaf displays selected BED files obtained from the RiceENCODE database [52], which contains tissue-specific chromatin accessibility, histone modification, and DNA methylation data, among others, obtained from high-throughput sequencing experiments.

#### Summary of results

The summary of results of the various analyses performed by a user is integrated into an interactive table and displayed on a dedicated summary page. The rows of the table correspond to the candidate genes, and the columns contain summary statistics such as the number of pathways containing the gene, the number of pathways or ontology terms associated with the coexpression network cluster to which the gene belongs, and the number of articles in which the gene was found based on our text-mining results. The rows of the table can be sorted based on multiple columns, allowing the user to prioritize the genes based on criteria they deem important.

### Demonstration of use case

We demonstrate the functionality and features of RicePilaf by applying it to a QTL analysis on yield under drought and a recent GWAS on preharvest sprouting.

#### Candidate genes underlying yield-under-drought QTLs in rice

We utilized RicePilaf to examine the genes underlying a large-effect QTL for improved rice yield under drought (qDTY12.1). Dixit et al. [53] undertook *in silico* characterization and quantitative PCR (qPCR) analyses of 53 intra-QTL candidate gene models underlying qDTY12.1 [54]. Candidate genes were based on the annotation of the Nipponbare reference genome, which was the best-annotated genome at that time.

RicePilaf includes genomes from circum-Aus N22 and circum-Basmati ARC 10497 subpopulations that are more closely related to the rice varieties used as donors to qDTY12.1, and hence we anticipate the identification of novel genes in the QTL that are not found in Nipponbare reference. Using the physical interval of qDTY12.1 in the Nipponbare reference genome (Chr12:15121175-18184336 bp, estimated from the physical positions of simple sequence repeats or SSRs used in the QTL mapping by Dixit et al. [53] and Mishra et al. [55]), a lift-over analysis was conducted against the N22 and ARC 10497 genomes. In total, 389 genes were found in this QTL interval in Nipponbare and 142 genes in the lift-over regions in N22. Among these, 109 genes are common to NB and N22 cultivars, while 28 genes are unique to N22 (Table 1). For ARC 10497 lift-over, 142 genes are in ARC 10497 lift-over regions, with 111 genes common to Nipponbare and ARC 10497. Twenty-four genes are unique to ARC (Table 2).

**Table 1:** Intra-QTL genes from the lift-over of QTL qDTY12.1 from Nipponbare that are unique to the N22 genome

N22 gene name	Description	UniProtKB/Swiss-Prot	OGI (Rice Gene Index ID)
*In the same chromosome*			
OsN22_12G011840	No known annotation	No known mapping	OGI:12042120
OsN22_12G011921			OGI:12042590
OsN22_12G012060			OGI:12043500
OsN22_12G012070			OGI:12043530
OsN22_12G012110			OGI:12043640
OsN22_12G012210			OGI:06025660
OsN22_12G012220			OGI:12043990
OsN22_12G012262			OGI:12046180
OsN22_12G012390			OGI:12046090
OsN22_12G012421			OGI:12046130
OsN22_12G012450			OGI:12018740
OsN22_12G012530			OGI:12046710
OsN22_12G012671			OGI:12047920
OsN22_12G012720			OGI:12048080
OsN22_12G013090			OGI:03099600
OsN22_12G013150			OGI:12049060
OsN22_12G013200			OGI:12049140
OsN22_12G013480	Disease resistance protein RPS2	Q42484	OGI:12050040
OsN22_12G012830	Wall-associated receptor kinase 3	Q9LMN8	OGI:12048340
OsN22_12G012870	Receptor-like protein EIX2	Q6JN46	OGI:12048400
*In different chromosomes*			
OsN22_01G004210	No known annotation	No known mapping	OGI:01011120
OsN22_06G029780			OGI:06087140
OsN22_09G011341			OGI:09045920
OsN22_06G017380			OGI:06052670
OsN22_10G003960	(S)-N-methylcoclaurine 3′-hydroxylase isozyme 1 (fragment)	Q9SP06	OGI:10016160
OsN22_09G011340	Amino acid permease 4	Q9FN04	OGI:09045880
OsN22_09G014850	UDP-glycosyltransferase 88B1	Q6VAA7	OGI:09054580
OsN22_02G022160	Sugar transport protein MST1	Q0JCR9	OGI:02062760

**Table 2:** Intra-QTL genes from the lift-over of qDTY12.1 from Nipponbare that are unique to the ARC 10497 genome

ARC 10497 gene name	Description	UniProtKB/Swiss-Prot	OGI (Rice Gene Index ID)
*In the same chromosome*			
OsARC_12g0011921	No known annotation	No known mapping	OGI:12038540
OsARC_12g0011930			OGI:12042120
OsARC_12g0011950			OGI:12042180
OsARC_12g0012021			OGI:12042810
OsARC_12g0012030			OGI:12042870
OsARC_12g0012120			OGI:12043530
OsARC_12g0012170			OGI:12043640
OsARC_12g0012240			OGI:12043840
OsARC_12g0012250			OGI:12014630
OsARC_12g0012350			OGI:12045300
OsARC_12g0012500			OGI:12046010
OsARC_12g0012590			OGI:12018740
OsARC_12g0012730			OGI:12046510
OsARC_12g0012880			OGI:12048080
OsARC_12g0013120			OGI:12048660
OsARC_12g0013270			OGI:03099600
OsARC_12g0013350			OGI:12049140
OsARC_12g0013640	Ent-sandaracopimara-8(14),15-diene synthase, chloroplastic	Q2QQJ5	OGI:12050140
OsARC_12g0012970	Wall-associated receptor kinase 4	Q9LMN6	OGI:12048340
OsARC_12g0013010	Receptor-like protein EIX2	Q6JN46	OGI:12048400
*In different chromosomes*			
OsARC_10g0006180	No known annotation	No known mapping	OGI:10026860
OsARC_06g0022101			OGI:06069780
OsARC_04g0000110			OGI:04000540
OsARC_10g0005060			OGI:10020180

The QTL qDTY12.1 was reported to interact with QTLs qDTY2.3 and qDTY3.2 [53], further enhancing yield-under-drought stress. Coexpression network analysis was conducted using the intra-QTL genes from qDTY12.1 and run with RiceNet v2 as the coexpression network, ClusterONE as the module detection algorithm, and 0.3 as the minimum cluster density for module detection.

**Figure 6:**
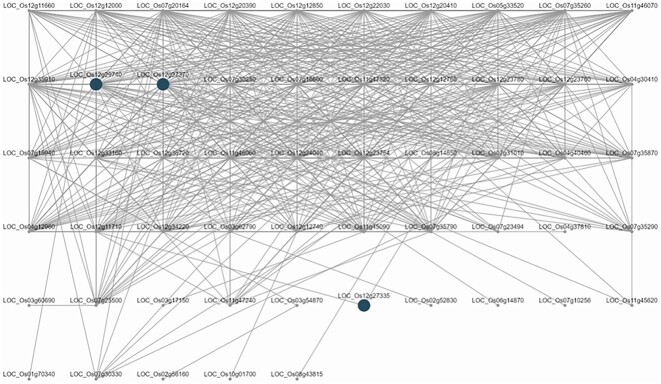
Coexpression network analysis using yield-under-drought QTL qDTY12.1 intra-QTL genes for module 347 that interact with intra-QTL genes for QTL qDTY2.3 module 347 includes intra-QTL gene (LOC_OS02g52830). qDTY12.1 was reported to interact with qDTY2.3 by Dixit et al. [53]. The shaded nodes indicate genes that fall within the physical interval of qDTY12.1 (Chr12:15121175-1818433).

**Figure 7:**
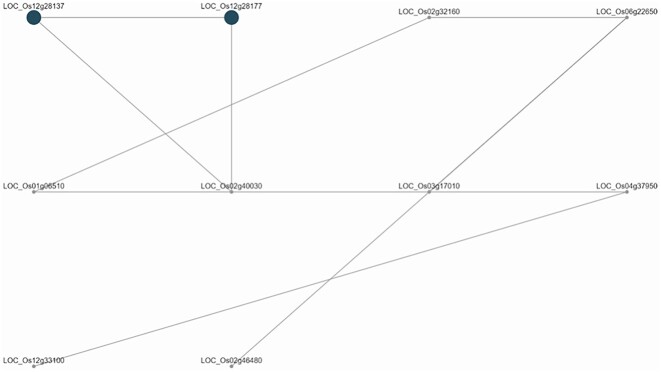
Coexpression network analysis using yield-under-drought QTL qDTY12.1 intra-QTL genes for module 111 that interact with intra-QTL genes for QTL qDTY2.3 module 111 includes intra-QTL gene LOC_Os02g46480. qDTY12.1 was reported to interact with qDTY2.3 by Dixit et al. [53]. The shaded nodes indicate genes that fall within the physical interval of qDTY12.1 (Chr12:15121175-1818433).

Results show that 3 of 2,608 discovered modules were enriched (adjusted *P* < 0.05). Two modules (namely, modules 347 and 111) were found to have intra-QTL genes from qDTY12.1 interacting with intra-QTL genes found in qDTY2.3. Module 347 (adjusted *P* = 0.02821) has 55 genes, and an interaction with qDTY2.3 intra-QTL gene LOC_OS02g52830 (lipase, putative, expressed) was reported. Module 111 (adjusted *P* = 0.02821) has 10 genes, of which an interaction with qDTY2.3 intra-QTL gene LOC_Os02g46480 (expressed protein) was reported. The interaction networks for these modules are shown in Figs. 6 and 7.

From these analyses done using RicePilaf, a targeted set of novel candidates from the 2 reference genomes from subpopulations that are more closely related to the QTL donor varieties were identified, which were not reported at the time of the initial studies. Two additional candidate genes from interacting QTL qDTY2.3 were also identified. These additional candidates can be used in future studies to further understand the mechanism of yield-under-drought stress across various drought-tolerant rice varieties and drought–tolerance QTL interactions at the gene level.

#### Post-GWAS analysis of preharvest sprouting

Preharvest sprouting (PHS) is a condition in which seeds lose dormancy and germinate prior to harvest, thus negatively affecting grain yield and quality [56]. A recent GWAS on PHS using a panel of 277 accessions representing temperate and tropical japonica and indica populations found the loci Chr01:1523,625-1770814 and Chr04:4662701-4670717 to be significantly associated with this trait [12].

##### Gene list and lift-over

We lifted over the PHS loci to the indica IR64 genome since the PHS GWAS by Lee et al. [12] contains indica accessions. We found 36 gene models overlapping the PHS loci in Nipponbare, of which 22 had orthologs in the corresponding IR64 intervals. Interestingly, of the genes unique to IR64, there were 3 whose Nipponbare orthologs were not contained in the original Nipponbare intervals (Table 3). These genes were not considered in the PHS GWAS by Lee et al. [12], demonstrating the benefit of lift-over. We included them in the 36 Nipponbare genes for further analysis.

**Table 3:** Genes unique to IR64 in the preharvest sprouting GWAS loci

Gene name	Description	UniProtKB/Swiss-Prot	Ortholog in Nipponbare
OsIR64_01g0001940	Bowman–Birk type bran trypsin inhibitor	A2WK50	LOC_Os01g03680
OsIR64_01g0001950	Putative cysteine-rich receptor-like protein kinase 33	Q9LDN1	LOC_Os01g03690
OsIR64_01g0002290	Mediator of RNA polymerase II transcription subunit 22a	Q9SA42	LOC_Os01g04110
OsIR64_01g0002000	No known annotation	No known mapping	No known orthologs
OsIR64_01g0002030			
OsIR64_01g0002040			
OsIR64_01g0002190			
OsIR64_01g0002200			
OsIR64_01g0002240			
OsIR64_01g0002260			
OsIR64_01g0002270			
OsIR64_01g0002280			

##### Coexpression network analysis

Out of 2,608 modules found by running ClusterONE on RiceNet v2 with the minimum cluster density set to 0.3, we found 39 modules that were enriched in the genes obtained in the previous step (adjusted *P* < 0.05). Without the 3 additional genes, there were 36 modules, further emphasizing the importance of RicePilaf’s lift-over feature. These 36 modules provide a narrower list of candidate genes possibly involved in PHS that could be experimentally tested.

Among the top 3 enriched modules—namely, modules 690 (adjusted *P* = 0.001214), 2425 (adjusted *P* = 0.001214), and 901 (adjusted *P* = 0.001405)—common enriched gene ontology terms include tryptophan biosynthetic process and serine-type carboxypeptidase (SCP) activity. Tryptophan has been reported to impact seed dormancy and PHS in wheat [57], and some SCPs and SCP-like proteins are known to be involved in the regulation of seed germination in rice and other crops [58–60]. In module 690, the phytohormone jasmonic acid—which, along with its derivatives, is related to seed dormancy and germination [61–63]—appears as an enriched trait ontology term.

In module 901, enriched gene ontology terms include the activities of β-glucosidase and β-amylase; certain genes belonging to these classes have been reported to be upregulated during pregermination and early germination, presumably for their role in starch degradation [64], which corroborates with starch and sucrose metabolism also appearing as an enriched pathway. Another pathway of interest in this module is the biosynthesis of various secondary plant metabolites such as coumarin, the ability of which to inhibit abscisic acid catabolism has been used to block PHS and vivipary in rice [65].

**Figure 8:**
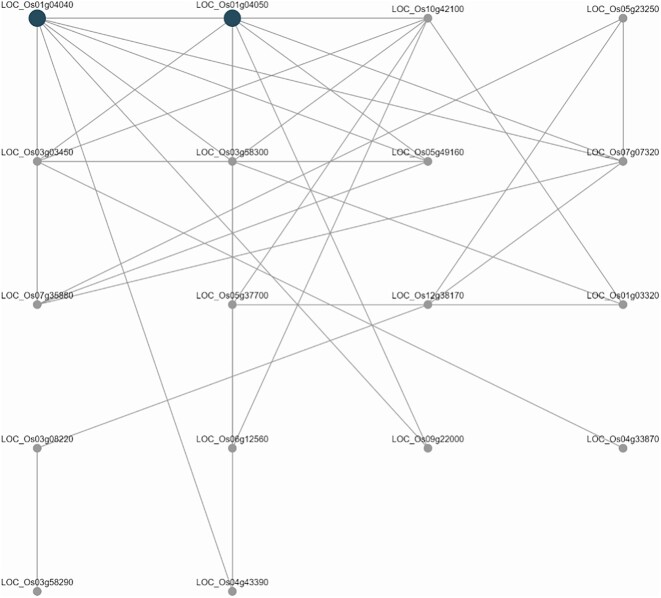
Coexpression network analysis of the loci Chr01:1523,625-1770814 and Chr04:4662701-4670717, known to be significantly associated with preharvest sprouting [12]. The graph is a module (module 690) that is enriched in ontology terms and pathways related to seed germination, dormancy, and vivipary, such as activities of β-glucosidase, β-amylase, starch and sucrose metabolism, and biosynthesis of coumarin. The shaded nodes indicate genes that fall within the specified loci.

Except for the starch and sucrose metabolism pathway, these aforementioned ontology terms and pathways were not reported in the PHS GWAS by Lee et al. [12], showing how RicePilaf’s coexpression network analysis can provide further functional insights into rice GWAS loci. The genes in the discovered modules (as in Fig. 8) can also be investigated experimentally for possible involvement in PHS and related biological processes.

To demonstrate the utility of RicePilaf providing multiple module detection algorithms in enriching post-GWAS analysis, we explored the enriched modules when the algorithm was set to FOX instead (with the weighted community clustering metric set to 0.05). Compared to when only the 36 gene models overlapping the PHS loci in Nipponbare were considered, the inclusion of the 3 Nipponbare orthologs from lift-over (Table 3) resulted in 10 additional enriched modules; in total, out of 4,416 discovered modules, 34 modules were found to be enriched.

Among the top 5 enriched modules, modules 1093 (adjusted *P* = 0.02398) and 1331 (adjusted *P* = 0.02398) in particular are enriched in germination- and growth-associated ontology terms related to the activity of key phytohormones (e.g., regulation of auxin biosynthetic process, auxin homeostasis, and gibberellic acid homeostasis), plant development (e.g., shoot system development and lateral root development), and metabolic activities (e.g., sucrose metabolic process and regulation of starch biosynthetic process). Plant embryo stage and seedling development stage also appear as enriched plant ontology terms in module 1331.

##### Enrichment in transcription factor binding sites

The top transcription factors whose binding sites significantly overlap with the PHS loci were CAMTA, FAR1, and ERF (each with an adjusted *P* of 0.1). These transcription factor families have been reported to be involved in biotic and abiotic stress response [66, 67].

## Limitations

RicePilaf integrates several existing tools and methods and depends on currently available datasets (Table 4) and thus carries the limitations inherent in those tools and data. For example, genomes and gene models are only available for the reference genomes currently available at the time of writing; however, we intend to update genomes periodically on the public site. Data on regulatory and coexpression networks in rice depend on the currently available data from a limited number of tissues and conditions.

**Table 4:** Summary of datasets used

Dataset type	Project, URL	Publication
Genome sequences, annotation, gene descriptions, and orthology maps of rice varieties	Rice Gene Index, https://riceome.hzau.edu.cn/	Yu et al. [24]
Coexpression network	RiceNet v2, https://www.inetbio.org/ricenet/	Lee et al. [41]
Coexpression network	Rice Combined Mutual Ranked Network (RCRN), https://doi.org/10.5061/dryad.zgmsbcc69	Zhao et al. [42]
Gene ontology annotations	Rice Annotation Project Database (RAP-DB), https://rapdb.dna.affrc.go.jp/	Sakai et al. [75]
Gene ontology annotations	agriGO v2.0, http://systemsbiology.cau.edu.cn/agriGOv2/	Tian et al. [43]
Gene, plant, and trait ontology annotations	Oryzabase, https://shigen.nig.ac.jp/rice/oryzabase/locale/change?lang=en	Kurata & Yamazaki [45]
Pathway maps	Kyoto Encyclopedia of Genes and Genomes (KEGG), https://www.genome.jp/kegg/	Kanehisa & Goto [44]
TF binding sites	PlantRegMap, http://plantregmap.gao-lab.org/	Tian et al. [50]
QTL from published literature	QTARO, http://qtaro.abr.affrc.go.jp/ accessed 2016-06	Yonemaru et al. [29]
Open chromatin	RiceENCODE, http://glab.hzau.edu.cn/RiceENCODE/	Xie et al. [52]

RicePilaf currently also lacks the means to incorporate tissue-specific gene expression information to narrow down the list of candidate genes, since currently available expression data have a limited range of tissues or use older technology that is not straightforward to integrate (e.g., [68, 69]). Similarly, epigenomic data such as chromatin accessibility and histone modification marks, which are known to be condition and tissue specific, are currently available for a very limited variety of tissues and come from only a few samples. In the lift-over functionality, there may be limitations related to possible multiplicity of alignment (in case of large segmental duplications). Currently, we force LAST to output only one-to-one alignments; thus, any homologous regions due to duplications are not possible to interrogate.

## Outlook

### Handling database updates

RicePilaf integrates information from multiple databases that are bound to see upgrades in the future. Additionally, in order for RicePilaf to respond quickly to user queries, we preprocess raw data from these databases to precompute information such as alignments, module detection, identification of enriched modules, ontology and pathway enrichment analysis, and annotations of PubMed abstracts. We incorporated the scripts for downloading and preprocessing into a Snakemake pipeline [70]. These scripts, along with all the necessary dependencies, are bundled into a Docker image (separate from the image for running the app), which can be downloaded from the code repository.

### Adding new features

RicePilaf follows a modular and extensible design that allows for easy addition and updating of features in the future. One key feature of immediate interest is collecting additional information from remote RESTful application programming interfaces (APIs). Databases that provide API access include UniProt [71], Gramene [72], and AgroLD [73]. Another key feature is including complementary data retrieval APIs like PyRice [28] to expand and facilitate a broader search of information.

## Conclusion

RicePilaf enables rice breeders and scientists to quickly cross-reference GWAS/QTL analysis results with a variety of rice databases. There have been several publications that identify potential QTLs and GWAS regions that remain poorly characterized for their specific mechanisms, and the overarching philosophy that drives this software development effort is the desire to solve this big unknown. This software platform is intended as a tool in order to understand many other QTLs’ functionality and figure out ways of dissecting the mechanistic granularities. Otherwise, we will just be building layers over layers without getting to the core. As an example, there are at least 3 other QTLs identified at the International Rice Research Institute now that operate as multigene QTLs. Hence, having an analysis pipeline for dissecting such regions for critical genes will add value as we and others discover more multigene QTLs in the future, especially in rice, mainly due to its compact genome. RicePilaf is easy to install, as it simply requires downloading a Docker image, along with the preprocessed dataset, and spinning up the container. It is also easy to use as it runs on a browser providing a user-friendly interface and a set of interactive web reports.

## Methods

### Lift-over

RicePilaf can translate GWAS/QTL regions on Nipponbare to genomic intervals of other accessions representing major Asian rice populations. Currently, the choice of genome as lift-over target includes (i) tropical Japonica Azucena, (ii) subtropical Japonica CHAO MEO, (iii) circum-Aus N22, (iv) indica IR64, (v) indica MH63, and (vi) circum-Basmati ARC 10497.

The lift-over from Nipponbare to a target genome is performed as follows. Pairwise whole-genome sequence alignment between Nipponbare and the target genome is precomputed using LAST as described in [74]. LAST produces a set of one-to-one local alignments (i.e., a base pair in Nipponbare aligns to at most 1 base pair in the target and vice versa). Additionally, there is no constraint for the alignments to be colinear, which allows for capturing complex inter- or intrachromosomal genome rearrangements. These precomputed alignments map Nipponbare genomic intervals to orthologous regions in the target. The set of gene models overlapping the target intervals is obtained from the genome annotations provided by the RGI [24]. The same project also provides orthologous gene groups, which can be used to compare GWAS/QTL gene sets across different accessions.

### Coexpression network analysis

RicePilaf integrates coexpression information in 3 stages: detection of modules (also known as communities or clusters), identification of modules enriched in the GWAS/QTL genes, and functional characterization of these modules by ontology and pathway enrichment analysis. We describe these steps in detail below.

#### Module/community detection

First, RicePilaf identifies modules of genes given a coexpression network; users can select either RiceNet v2 [41] or the Rice Combined Mutual Ranked Network (RCRN) [42]. For RiceNet v2, the coexpression network used is the component network derived from the coexpression of *Oryza sativa* genes across microarray experiments. For RCRN, the integrated network is used.

Since genes can possibly be involved in multiple biological functions or processes [76], the supported module detection algorithms allow for overlapping modules (i.e., a given gene may belong to multiple modules). To this end, RicePilaf provides a selection of 4 algorithms: (i) ClusterONE [77], (ii) COACH [78], (iii) DEMON [79], and (iv) FOX [80].

#### Identification of enriched modules

From among the detected gene modules, RicePilaf performs overrepresentation analysis to identify which modules are statistically enriched in view of the coexpression network and the GWAS/QTL-implicated genes. To this end, a 2 × 2 contingency table is constructed, with all the genes across the detected modules comprising the background gene set. The columns count the number of genes implicated by GWAS/QTL (versus those that are not implicated), and the rows count the number of genes present in the module being tested (versus those that are not present). A 1-tailed Fisher’s exact test is then applied, followed by multiple-testing correction via the Benjamini–Hochberg method [81]. A module is considered enriched if its adjusted *P* value is less than 0.05.

#### Functional characterization via ontology and pathway enrichment analysis

The likely biological functions of the enriched modules are inferred by performing enrichment analysis across several ontology and pathway databases. For the ontology enrichment analysis, RicePilaf displays results for 3 sets of ontologies: (i) gene ontology, (ii) trait ontology, and (iii) plant ontology. Gene ontology annotations—which cover cellular components, molecular functions, and biological processes—are aggregated from the Rice Annotation Project Database (RAP-DB) [75], agriGO v2.0 [43], and Oryzabase [45]. Trait and plant ontology annotations—which focus on phenotypic attributes—are obtained from Oryzabase [45].

For identifying enriched pathways, RicePilaf supports both overrepresentation analysis via clusterProfiler [82] and topology-based analysis via Pathway-Express [83] and Signaling Pathway Impact Analysis (SPIA) [84]. Pathway maps are obtained from the KEGG [44]; accessions are mapped to KEGG identifiers using the R package riceidconverter [85] and mapping tables from RAP-DB [75]. An ontology term or pathway is considered enriched if its adjusted *P* value after Benjamini–Hochberg correction [81] is less than 0.05.

### Enrichment of regulatory features

RicePilaf determines if the input GWAS/QTL intervals are enriched for binding sites of TFs. This is done by first computing overlaps between GWAS/QTL genomic intervals and predicted binding sites of a TF using Pybedtools [86, 87]. For TFs that have a nonempty intersection, the statistical significance of the overlap is computed using MCDP2 [88], and multiple testing across multiple TFs is accounted for by Benjamini–Hochberg correction of the significance values [81]. Binding site information of almost 250 TFs is obtained from PlantRegMap [50]. For each TF, PlantRegMap provides several sets of predicted binding sites depending on how the prediction was performed—(i) simple motif scanning using FIMO [89] or (ii) motif scanning paired with conserved element information [50] or (iii) using the FunTFBS method [50]—and what target sequence was used: whole genome versus promoter region defined as −500/+100 bp of the transcription start site. RicePilaf exposes these choices to the user.

### Text mining

Around 17,000 scientific abstracts were retrieved from PubMed by using a curated list of PubMed identifiers provided by the Oryzabase database [45]. This list provides manually checked PubMed entries related to rice genomics. A natural language processing pipeline was written using Python to extract named entities from these abstracts. This pipeline combines the HunFLAIR named entity recognition tagger [90] with spaCy [91], Natural Language Toolkit (NLTK) [92], and other libraries. It identifies 4 types of named entity annotations: gene names (e.g., “OsMAPK2” or “MOC1”), species (e.g., “Oryza sativa” or “Magnaporthe oryzae”), chemicals (e.g., “gibberellic acid” or “nitrogen”), and disease or phenotype (e.g., “blast disease” or “sheath blight disease”). In total, 351,003 annotations of 63,591 distinct named entities were identified from these PubMed abstracts and titles.

RicePilaf performs quick searches on these annotations to retrieve gene names associated with diseases, phenotypes, chemicals, or species. The retrieved genes can be added to the coexpression network and regulatory enrichment analyses, thus further enriching the post-GWAS analysis.

### A summary of datasets used

A summary of the datasets used in RicePilaf is presented in Table 4.

## Availability of Source Code and Requirements

- Project name: RicePilaf
- Project homepage: The source code and URLs to publicly accessible running instances of RicePilaf are available at  https://github.com/bioinfodlsu/rice-pilaf.
- Operating system(s): Platform-independent
- Programming language: Python Dash and Flask, R, Snakemake
- Other requirements: web browser, Docker (for installing locally or deploying to a server)
- License: MIT License
- SciCrunch RRID:SCR_024945
- bio.tools URL: https://bio.tools/ricepilaf

## Abbreviations

API: application programming interface; GWAS: genome-wide association study; KEGG: Kyoto Encyclopedia of Genes and Genomes; LD: linkage disequilibrium; NLTK: Natural Language Toolkit; PHS: preharvest sprouting; QTL: quantitative trait loci; RAP-DB: Rice Annotation Project Database; RCRN: Rice Combined Mutual Ranked Network; RGI: Rice Gene Index; SCP: serine-type carboxypeptidase; SNP: single-nucleotide polymorphism; SSR: simple sequence repeat; TF: transcription factor.

## Supplementary Material

giae013_GIGA-D-23-00310_Original_Submission

giae013_GIGA-D-23-00310_Revision_1

giae013_Response_to_Reviewer_Comments_Original_Submission

giae013_Reviewer_1_Report_Original_Submission Mao Suganami -- 11/20/2023 Reviewed

giae013_Reviewer_2_Report_Original_Submission Weilong Kong -- 11/23/2023 Reviewed

## Data Availability

The URL to the dataset required for running the app can be found in the project’s repository. This dataset has been generated from publicly available datasets described in Table 4 using the Snakemake pipeline available in the project’s code repository. Other supporting materials are available in Software Heritage Archive [93].
