# Supplementary material for: RicePilaf: a post-GWAS/QTL dashboard to integrate pangenomic, coexpression, regulatory, epigenomic, ontology, pathway, and text-mining information to provide functional insights into rice QTLs and GWAS loci
Source: Gigascience. 2024 Jun 4;13:giae013. doi: 10.1093/gigascience/giae013 (PMC11148593; doi:10.1093/gigascience/giae013)
Supplement: giae013_GIGA-D-23-00310_Revision_1 [file giae013_giga-d-23-00310_revision_1.pdf]

# GigaScience

# RicePilaf: a post-GWAS/QTL dashboard to integrate pangenomic, co-expression, regulatory, epigenomic, ontology, pathway, and text-mining information to provide functional insights into rice QTLs and GWAS loci

--Manuscript Draft--

| | |
|---|---|
| **Manuscript Number:** | GIGA-D-23-00310R1 |
| **Full Title:** | RicePilaf: a post-GWAS/QTL dashboard to integrate pangenomic, co-expression, regulatory, epigenomic, ontology, pathway, and text-mining information to provide functional insights into rice QTLs and GWAS loci |
| **Article Type:** | Technical Note |
| **Funding Information:** | Rural Development Administration (PJ016405) — Dr. Ji-Ung Jeung |
| **Abstract:** | Background: As the number of genome-wide association studies (GWAS) and quantitative trait loci (QTL) mappings in rice continues to grow, so does the already-long list of genomic loci associated with important agronomic traits. Typically, loci implicated by GWAS/QTL analysis contain tens to hundreds to thousands of SNPs/genes, not all of which are causal, and many of which are in non-coding regions. Unraveling the biological mechanisms that tie the GWAS regions and QTLs to the trait of interest is challenging, especially since it requires collating functional genomics information about the loci from multiple, disparate data sources.<br>Results: We present RicePilaf, a web app for post-GWAS/QTL analysis, which performs a slew of novel bioinformatics analyses to cross reference GWAS results and QTL mappings with a host of publicly available rice databases. In particular, it integrates (i) pangenomic information from high-quality genome builds of multiple rice varieties, (ii) co-expression information from genome-scale co-expression networks, (iii) ontology and pathway information, (iv) regulatory information from rice transcription factor databases, (v) epigenomic information from multiple high-throughput epigenetic experiments, and (vi) text-mining information extracted from scientific abstracts linking genes and traits. We demonstrate the utility of RicePilaf by applying it to analyze GWAS peaks of pre-harvest sprouting and genes underlying yield-under-drought QTLs.<br>Conclusions: RicePilaf enables rice scientists and breeders to shed functional light on their GWAS regions and QTLs, and provides them with a means to prioritize SNPs/genes for further experiments. The source code, a Docker image, and a demo version of RicePilaf are publicly available at https://github.com/bioinfodlsu/rice-pilaf. |
| **Corresponding Author:** | Kenneth McNally<br>International Rice Research Institute<br>Los Baños, Laguna, PHILIPPINES |
| **Corresponding Author Secondary Information:** | |
| **Corresponding Author's Institution:** | International Rice Research Institute |
| **Corresponding Author's Secondary Institution:** | |
| **First Author:** | Anish M.S. Shrestha |
| **First Author Secondary Information:** | |
| **Order of Authors:** | Anish M.S. Shrestha |
| | Mark Edward M. Gonzales |
| | Phoebe Clare L. Ong |
| | Pierre Larmande |
| | Hyun-Sook Lee |
| | Ji-Ung Jeung |
| | |

Powered by Editorial Manager® and ProduXion Manager® from Aries Systems Corporation

| | |
|---|---|
| | Ajay Kohli |
| | Dmytro Chebotarov |
| | Ramil P Mauleon |
| | Jae-Sung Lee |
| | Kenneth L. McNally |
| **Order of Authors Secondary Information:** | |
| **Response to Reviewers:** | Dear Mr. Edmunds,<br><br>We are pleased to submit our revised Technical note entitled “RicePilaf: a post-GWAS/QTL dashboard to integrate pangenomic, co-expression, regulatory, epigenomic, ontology, pathway, and text-mining information to provide functional insights into rice QTLs and GWAS loci” for your consideration. We would like to thank the editors and reviewers for their constructive comments. Based on their comments, we have implemented a major feature update of the software and updated the manuscript to reflect the changes. Changes in the manuscript are marked in red. With these, we have addressed almost all the issues raised, the only exception being a suggestion from Reviewer 1 to include the gene expression database RiceXPro which was not possible due to missing mapping information between microarray spots and RGAP gene IDs. A detailed point-by-point response follows.<br><br>Thank you for considering our submission.<br><br>Sincerely,<br>Kenneth L. McNally<br>IRRI, Philippines<br><br><br>Comments from the editor:<br><br>Please register Ricepilaf in the bio.tools and SciCrunch.org databases to receive RRID (Research Resource Identification Initiative ID) and biotoolsID identifiers, and include these in your manuscript. Computational workflows should be registered in workflowhub.eu and the DOIs cited in the relevant places in the manuscript. These will facilitate tracking, reproducibility and re-use of your tool.<br><br>Response:<br>We have registered RicePilaf in the bio.tools and SciCrunch.org databases. The bio.tools URL and SciCrunch RRID have been added to the Availability of source code and requirements section of the manuscript.<br><br>Comments from Reviewer 1:<br><br>1) I suggest that gene expression information databases such as RiceXPro (https://ricexpro.dna.affrc.go.jp/) and Transcriptome ENcyclopedia Of Rice (https://tenor.dna.affrc.go.jp/) should also be made available. By referring to organ-specific expression information of genes, it could be possible to narrow down the causative genes.<br><br>We agree with the reviewer that having organ/tissue-specific gene expression information would help narrow down the candidate gene list from GWAS on traits that are organ/tissue-specific. We had discussed this issue in the Limitations section. We have expanded the discussion to include the RiceXPro and TENOR databases.<br><br>However we were not able to easily incorporate the two databases into our app. RiceXPro uses Agilent Microarray, and the NCBI GEO dataset does not provide a mapping of the microarray spot to RAP or RGAP7 IDs, making it not possible to integrate the results in NCBI GEO used in RiceXPro to the RicePilaf. On the other hand, the data available for download from the TENOR dataset contains only expression data from two organs: root and shoot and not comprehensive enough to build a candidate gene filter in RicePilaf. While in theory, we could utilize the TENOR dataset to instead build a co-expression network, we already employ two similarly built |

| | |
|---|---|
| | co-expression networks (RiceNet v2 and RCRN) in the app.<br><br>2) This paper presents the results of the analysis of two cases using RicePilaf. However, the paper only describes the results and discussion of each analysis section, and lacks the process of integrating these results and narrowing them down to the candidate genes. Given that the goal of this paper is to obtain functional insights by multiplying multiple analysis sections, the route to this goal should be more clearly presented.<br><br>As a response to this comment, we have implemented a major feature update to integrate the results and to allow users to narrow down candidate genes.<br><br>The summary of results of the various analyses performed by the user is integrated into an interactive table and displayed in the Summary page. Rows of the table correspond to the candidate genes and columns contain summary statistics such as the number of pathways that contain the gene, the number of pathways/ontology terms that are associated with co-expression network cluster the gene belongs to, the number of articles the gene was found in our text-mining results, etc. The rows of the table can be sorted based on multiple columns, allowing the user to prioritize the genes based on criteria they deem important.<br><br>Comments from Reviewer 2:<br><br>1) As far as I know, there are already a lot of databases on rice genomes, transcriptomes, and epigenomics. What are the advantages of this platform? Please provide a specific picture statistic for those data included, refer to Figure 1A of https://doi.org/10.1093/nar/gkad701<br><br>RicePilaf is not a database, rather a platform to integrate information from various existing databases and provide tools that are lacking in other databases. One of the strengths of the app is that the entry point to it is a GWAS/QTL region, something other platforms do not directly address.<br><br>As suggested, we have added a figure (Figure 1 in the manuscript), which provides a graphical abstract of the databases used in the app. Similar information is also available in Table 4, which provides a summary of the databases used.<br><br>2) Please add some citations of existing rice star databases and compare the advantages of this data platform with these existing databases.<br><br>The databases that the tool uses  are summarized in Table 4. Citations and URLs are provided in the table.<br><br>To reiterate, RicePilaf is not a database, rather a platform to integrate information from various existing databases. One of the strengths of the app is that the entry point to it is a GWAS/QTL region, something other platforms do not directly address.<br><br>3) The results of some existing quantitative traits are lacking, such as anaerobic germination (10.1002/fes3.354) and morphological traits (10.1111/tpj.15705 and 10.3389/fpls.2022.995634)<br><br>We have added the three citations.<br><br>4) In the abstract, "not all of which are causal, and many of which are in non-coding regions." Non-coding region variants are not necessarily not causal mutations, and it is suggested that this should be changed to "there are some false-positive results".<br><br>The sentence does not imply that non-coding region variants are not causal. Hence, we have not edited the statement. |
| **Additional Information:** | |
| **Question** | **Response** |
| Are you submitting this manuscript to a | No |

*Powered by Editorial Manager® and ProduXion Manager® from Aries Systems Corporation*

| | |
|---|---|
| special series or article collection? | |
| **Experimental design and statistics**<br><br>Full details of the experimental design and statistical methods used should be given in the Methods section, as detailed in our Minimum Standards Reporting Checklist. Information essential to interpreting the data presented should be made available in the figure legends.<br><br>Have you included all the information requested in your manuscript? | Yes |
| **Resources**<br><br>A description of all resources used, including antibodies, cell lines, animals and software tools, with enough information to allow them to be uniquely identified, should be included in the Methods section. Authors are strongly encouraged to cite Research Resource Identifiers (RRIDs) for antibodies, model organisms and tools, where possible.<br><br>Have you included the information requested as detailed in our Minimum Standards Reporting Checklist? | Yes |
| **Availability of data and materials**<br><br>All datasets and code on which the conclusions of the paper rely must be either included in your submission or deposited in publicly available repositories (where available and ethically appropriate), referencing such data using a unique identifier in the references and in the “Availability of Data and Materials” section of your manuscript.<br><br>Have you have met the above requirement as detailed in our Minimum Standards Reporting Checklist? | Yes |

Powered by Editorial Manager® and ProduXion Manager® from Aries Systems Corporation

Click here to access/download;LaTeX - Main Document (TeX file);main.tex

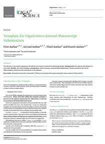

*GigaScience*, 2023, 1–12

doi: xx.xxxx/xxxx
Manuscript in Preparation
Technical Note

TECHNICAL NOTE

# RicePilaf: a post-GWAS/QTL dashboard to integrate pangenomic, co-expression, regulatory, epigenomic, ontology, pathway, and text-mining information to provide functional insights into rice QTLs and GWAS loci

Anish M.S. Shrestha[1,4,†,‡], Mark Edward M. Gonzales[1,†], Phoebe Clare L. Ong[1,†], Pierre Larmande[2], Hyun-Sook Lee[3], Ji-Ung Jeung[3], Ajay Kohli[4], Dmytro Chebotarov[4], Ramil P. Mauleon[4], Jae-Sung Lee[4] and Kenneth L. McNally[4,*]

[1]Bioinformatics Lab, Advanced Research Institute for Informatics, Computing and Networking, College of Computer Studies, De La Salle University, Manila, Philippines and [2]DIADE, Univ Montpellier, Cirad, IRD, Montpellier, France and [3]National Institute of Crop Science, 181 Hyeoksin-ro, Iseo-myeon, Wanju-gun, Jeollabuk-do, Republic of Korea (55365) and [4]International Rice Research Institute, DAPO Box 7777, Metro Manila 1301, Philippines

[*]K.McNally@irri.org
[‡]anish.shrestha@dlsu.edu.ph
[†]Contributed equally.

ORCIDs:
Anish M S Shrestha [0000-0002-9192-9709]; Mark Edward M Gonzales [0000-0001-5050-3157]; Phoebe Clare L Ong [0009-0004-7982-7314]; Pierre Larmande [0000-0002-2923-9790]; Hyun-Sook Lee [0000-0002-1959-6209]; Ji-Ung Jeung [0000-0002-7578-2081]; Ajay Kohli [0000-0002-7325-5798]; Dmytro Chebotarov [0000-0003-1351-9453]; Ramil P Mauleon [0000-0001-8512-144X]; Jae-Sung Lee [0000-0002-3644-4901]; Kenneth L McNally [0000-0002-9613-5537].

## Abstract

**Background:** As the number of genome-wide association studies (GWAS) and quantitative trait loci (QTL) mappings in rice continues to grow, so does the already-long list of genomic loci associated with important agronomic traits. Typically, loci implicated by GWAS/QTL analysis contain tens to hundreds to thousands of SNPs/genes, not all of which are causal, and many of which are in non-coding regions. Unraveling the biological mechanisms that tie the GWAS regions and QTLs to the trait of interest is challenging, especially since it requires collating functional genomics information about the loci from multiple, disparate data sources.
**Results:** We present RicePilaf, a web app for post-GWAS/QTL analysis, which performs a slew of novel bioinformatics analyses to cross-reference GWAS results and QTL mappings with a host of publicly available rice databases. In particular, it integrates *(i)* pangenomic information from high-quality genome builds of multiple rice varieties, *(ii)* co-expression information from genome-scale co-expression networks, *(iii)* ontology and pathway information, *(iv)* regulatory information from rice transcription factor databases, *(v)* epigenomic information from multiple high-throughput epigenetic experiments, and *(vi)* text-mining information extracted from scientific abstracts linking genes and traits. We demonstrate the utility of RicePilaf by applying it to analyze GWAS peaks of pre-harvest sprouting and genes underlying yield-under-drought QTLs.
**Conclusions:** RicePilaf enables rice scientists and breeders to shed functional light on their GWAS regions and QTLs, and provides them with a means to prioritize SNPs/genes for further experiments. The source code, a Docker image, and a demo version of RicePilaf are publicly available at `https://github.com/bioinfodlsu/rice-pilaf`.

**Key words**: rice; GWAS; QTL analysis; post-GWAS; co-expression network; transcription factor binding; text mining

**Key Points**

- Quantitative trait loci (QTL) analysis and genome-wide association studies (GWAS) in rice have identified a large number of loci-trait associations.
- Making sense of GWAS/QTL mapping results is challenging due to the large number of genes being implicated and the complex patterns of interactions among genes to produce a trait.
- RicePilaf cross-references GWAS/QTL-mapping results with multiple data sources on rice to provide insights into GWAS regions and QTLs.
- RicePilaf is free, open-source, and containerized. It can be run locally on a browser or can be set up as a web service.

## Background

Rice is a global food staple feeding half of humanity. To address the dual concerns of meeting the demands of a growing world population while minimizing contribution to climate change, scientists and breeders are continually seeking genetic sources for high-yield, sustainable, and robust rice varieties [1]. Paramount to this task are the identification and elucidation of the genetic and molecular basis of agronomically important traits.

Genome-wide association studies (GWAS) and quantitative trait locus (QTL) analysis in rice have identified a multitude of genetic loci influencing a wide range of important traits. Recent examples include GWAS/QTL analysis on variations in environmental stress responses (e.g., cold tolerance [2], heat tolerance [3, 4], and anaerobic germination [5, 6]), disease tolerance (e.g., blast [7]), mineral contents (e.g., cadmium accumulation [8]), morphological traits (e.g., plant height [9], grain weight [10], and grain size and panicle length [11]), and other yield-related traits (e.g., pre-harvest sprouting [12] and seed longevity [13]). This list is only expected to grow given the immense interest in the genetics of rice traits and the easy availability of genotyping resources covering millions of SNPs across thousands of rice varieties [14].

The biological interpretation of statistical loci-trait associations is challenging due to a number of reasons. First, a typical GWAS can implicate hundreds to thousands of SNPs due to high linkage disequilibrium (LD) — estimates of LD extend to hundreds of kilobases for some rice populations [15, 16]. QTL mappings and GWAS peaks likewise may have tens to hundreds of underlying genes. Not all of the SNPs/genes in the reported loci will be causal, requiring a mechanism to narrow down the candidate list. Next, complex traits are likely influenced by multiple SNPs/genes, which individually are only able to explain a small amount of variation. Teasing out biological meaning therefore requires taking into account co-expression and co-regulation patterns of groups of genes.

Furthermore, there might be many associated SNPs that are in intergenic and non-coding regions, given that a majority of SNPs in genotyping assays and genotype databases do not lie inside gene models [17, 18]. This requires incorporating regulatory information in the post-GWAS analysis. Lastly, since SNP genotypes are typically called against the Nipponbare reference, GWAS/QTL loci are typically reported only in Nipponbare coordinates, even though several high-quality genome assemblies of a variety of accessions are now available. Given that a large number of between-population genomic variations have been reported [14], a pangenomic view of gene sets implicated by GWAS/QTL mapping is necessary.

Thus, the post-GWAS/QTL mapping task of prioritizing genes or identifying biological mechanisms that link them to the phenotype requires the integration of GWAS/QTL mapping results with genomic information from a host of other data sources. While tools for computational post-GWAS analysis have been reported for other species (e.g. [19, 20]), there is a limited number of tools dedicated to rice, one of which is Rice Galaxy [21] (now folded into CropGalaxy [22]), which utilizes genome position information and lift-over across a few rice genomes.

Here we report RicePilaf, a web app for post-GWAS/QTL analysis that integrates rice GWAS/QTL mapping results with pangenomic, co-expression, epigenomic, ontology, pathway, regulatory, and literature-mining information coming from various data sources, and produces interactive web reports allowing users to, for example, search and sort data tables, move and click network nodes to display information on pertinent genes, and download the analysis results in text format. It is built using the Python-based Dash and Flask frameworks. All dependencies are bundled into a Docker image; hence, it works on any of the major operating systems. It can be run locally on a web browser or provided as a web service. It is free and open-source. Further details of the software are provided in the Availability of source code and requirements section. We demonstrate the utility of the software using recent GWAS/QTL analysis on two key traits connected to modern rice cultivation: yield-under-drought and pre-harvest sprouting.

## Results and discussion

### RicePilaf overview

RicePilaf takes in as input a set of genomic intervals (Figure 2), obtained from a QTL analysis or from clumping of LD-linked statistically significant SNPs from a GWAS, e.g., computed by the LD-clumping procedure of PLINK [23]. It performs a series of novel bioinformatics analyses on the input intervals, which we overview here and describe in more detail in the Methods section.

#### *Gene list*

RicePilaf begins by retrieving the gene models overlapping the input intervals in the Nipponbare reference. For each gene model, it provides the following: *(i)* gene description and orthology information obtained from Rice Gene Index (RGI) [24]; *(ii)* protein, protein domains, and protein family information from UniProt [25], InterPro [26], and Pfam [27], obtained by automated queries using PyRice [28]; and *(iii)* scientific literature associating the gene to traits, obtained from QTARO [29] and our in-house text-mined dataset.

#### *Lift-over*

Nipponbare serves as the gold-standard reference genome sequence and genomic coordinate system. Given that genotype calls are made against the Nipponbare reference, GWAS/QTL mapping results are reported in Nipponbare coordinates. Recently, high-quality genome builds of several accessions have been pub-

**Compiled on:** March 12, 2024.
Draft manuscript prepared by the author.

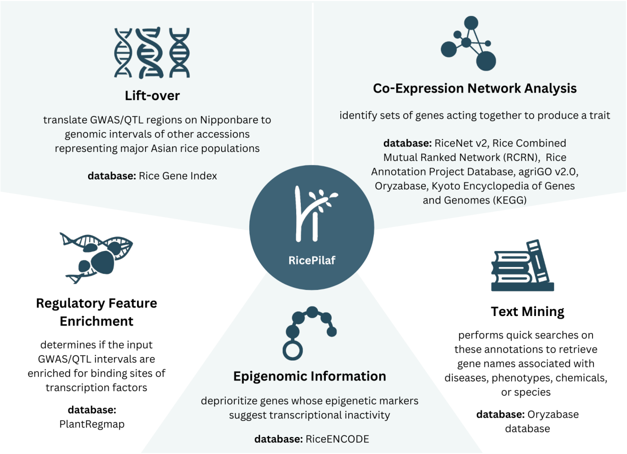


**Figure 1.** RicePilaf graphical abstract. RicePilaf crosses GWAS/QTL-mapping results with multiple data sources on rice. A summary of databases used in RicePilaf is summarized in Table 4.

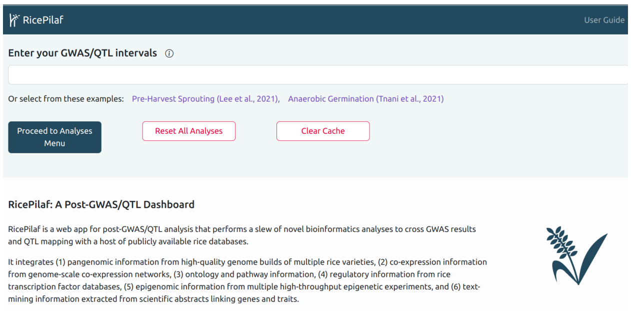


**Figure 2.** A screenshot of the input interface.

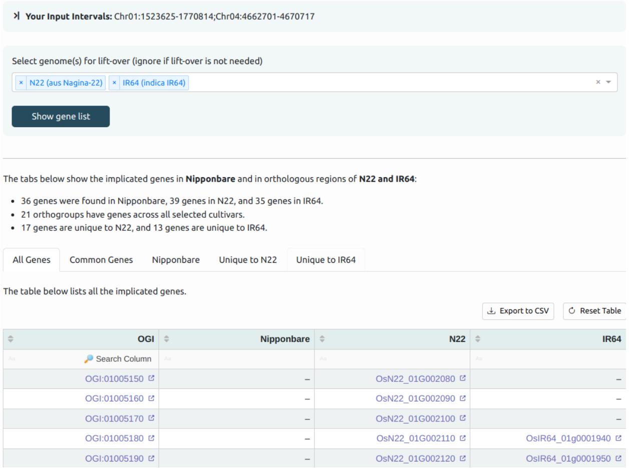


**Figure 3.** A screenshot showing an example of the result of lift-over from Nipponbare coordinates to another genome, IR64 in this case.

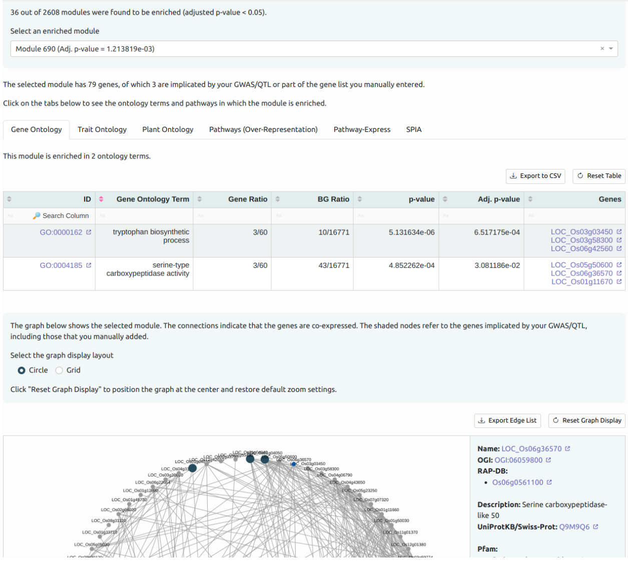


**Figure 4.** A screenshot showing an example of co-expression network analysis in RicePilaf.

lished [30, 31, 32]. Comparative genomics has revealed an abundance of duplications, deletions, insertions, inversions, and translocations during the evolution of rice [14, 33, 34].

For GWAS/QTL analysis on populations that are not derived from or include Nipponbare, by relying only on its genome and its annotation, we likely will miss genes and regulatory features linked to the phenotype of interest. Examples of genes of agronomic importance that are absent in Nipponbare (but present in indica or aus) include *Sub1* [35].

RicePilaf can lift over the intervals in the Nipponbare reference coordinates to several other recently published genomes, representing major rice populations. Using the Rice Gene Index [24] database, it retrieves the genes overlapping the lifted-over intervals and their orthologs. This pangenomic view of gene sets may be useful if, for example, the GWAS/QTL mapping is on an accession that is closer to a genome other than Nipponbare (Figure 3).

### *Co-expression network analysis*

Complex traits are influenced by hundreds of SNPs/genes which individually are only able to explain a small amount of variation in the trait. Genes with the same or similar biological functions or involved in the same pathway are likely to be also co-expressed [36, 37, 38]. Co-expression networks provide a means to identify sets of genes acting together to produce a trait. An additional benefit of a co-expression network is that for genes with poor annotations or unknown functions, their membership in a dense subnetwork containing well-characterized genes might be a way to uncover incomplete functional information. Furthermore, co-expression networks have been used for post-GWAS analysis in a number of plants and animals [20, 39, 40].

To identify genes that may be acting collectively to result in a trait, RicePilaf searches rice co-expression networks, RiceNet v2 [41] and RCRN [42], for modules (communities or clusters) of genes that are statistically enriched in the genes overlapping the input intervals. Functional characterization of the modules is done via enrichment analysis against several ontology and pathway databases from agriGO [43], KEGG [44], and Oryzabase [45] (Figure 4).

### *Regulatory feature enrichment*

A majority of rice SNPs in genotyping assays and genotype databases are located in non-coding regions [18, 17, 14]. For example, the 3,000 Rice Genomes Project found twice as many SNPs in intergenic regions than within genes [46]. Unsurprisingly GWAS/QTL mappings also report many non-coding trait-associated variants. It is likely that these influence the activity of regulatory elements. One possible causal link is that variants could alter transcription factor binding affinity leading to changes in the expression of target genes, ultimately resulting in phenotypic variation [47]. Post-GWAS tools for human data that overlap GWAS results with regulatory features such as TF binding sites, chromatin accessibility, and histone marks [48, 49] have been previously reported.

To investigate variants that might be affecting the binding activity of transcription factors, RicePilaf searches for transcrip-

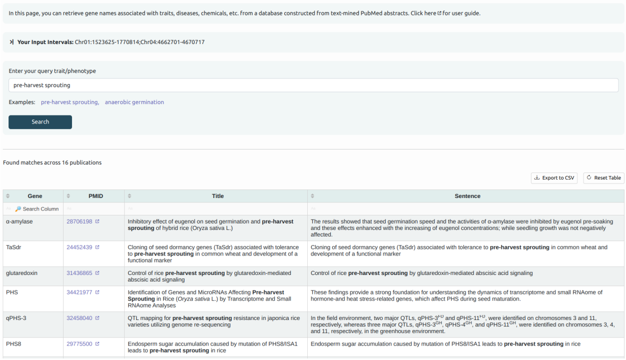


**Figure 5.** A screenshot showing an example of the result of searching against a text-mining derived database containing information from PubMed abstracts.

tion factors whose known/predicted binding sites provided by PlantRegMap [50] significantly overlap with the input intervals.

### *Text mining*

The gene list provided for the co-expression and regulatory enrichment analyses can be supplemented by genes retrieved from the pangenome liftover and from querying our in-house dataset obtained by text mining PubMed abstracts on rice gene-trait associations. Additionally, the same text-mining dataset is used to find scientific literature related to the genes overlapping the input interval.

### *Epigenomic information*

For traits that are tissue-specific, it may be desirable to deprioritize genes whose epigenetic markers suggest transcriptional inactivity. Using the embeddable Integrative Genomics Viewer [51], RicePilaf displays selected BED files obtained from the RiceENCODE database [52], which contains tissue-specific chromatin accessibility, histone modification, and DNA methylation data among others, obtained from high-throughput sequencing experiments.

### *Summary of results*

The summary of results of the various analyses performed by a user is integrated into an interactive table and displayed on a dedicated summary page. The rows of the table correspond to the candidate genes, and the columns contain summary statistics such as the number of pathways containing the gene, the number of pathways or ontology terms associated with the co-expression network cluster to which the gene belongs, and the number of articles in which the gene was found based on our text-mining results. The rows of the table can be sorted based on multiple columns, allowing the user to prioritize the genes based on criteria they deem important.

## Demonstration of use case

We demonstrate the functionality and features of RicePilaf by applying it to a QTL analysis on yield-under-drought and a recent GWAS on pre-harvest sprouting.

### *Candidate genes underlying yield-under-drought QTLs in rice*

We utilized RicePilaf to examine the genes underlying a large-effect QTL for improved rice yield under drought (qDTY12.1). Dixit *et al.* [53] undertook *in silico* characterization and qPCR analyses of 53 intra-QTL candidate gene models underlying qDTY12.1 [54]. Candidate genes were based on the annotation of the Nipponbare reference genome which was the best-annotated genome at that time.

RicePilaf includes genomes from circum-Aus N22 and circum-Basmati ARC 10497 subpopulations that are more closely related to the rice varieties used as donors to qDTY12.1, hence we anticipate the identification of novel genes in the QTL that are not found in

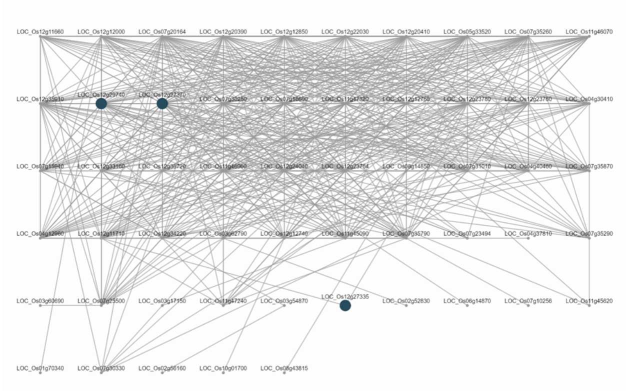

**Figure 6.** Co-expression network analysis using yield-under-drought QTL qDTY12.1 intra-QTL genes for Module 347 that interact with intra-QTL genes for QTL qDTY2.3 Module 347 includes intra-QTL gene (LOC_OS02g52830). qDTY12.1 was reported to interact with qDTY2.3 by Dixit *et al.* [53]. The shaded nodes indicate genes that fall within the physical interval of qDTY12.1 (Chr12:15121175-1818433).

Nipponbare reference. Using the physical interval of qDTY12.1 in the Nipponbare reference genome (Chr12:15121175-18184336 bp, estimated from the physical positions of simple sequence repeats or SSRs used in the QTL mapping by Dixit *et al.* [53] and Mishra *et al.* [55]), a lift-over analysis was conducted against the N22 and ARC 10497 genomes. 389 genes were found in this QTL interval in Nipponbare, and 142 genes in the lift-over regions in N22. Among these, 109 genes are common to NB and N22 cultivars, while 28 genes are unique to N22 (Table 1). For ARC 10497 lift-over, 142 genes in ARC 10497 lift-over regions, with 111 genes common to Nipponbare and ARC 10497. 24 genes are unique to ARC (Table 2).

The QTL qDTY12.1 was reported to interact with QTLs qDTY2.3 and qDTY3.2 [53], further enhancing yield-under-drought stress. Co-expression network analysis was conducted using the intra-QTL genes from qDTY12.1 and run with RiceNet v2 as the co-expression network, ClusterONE as the module detection algorithm, and 0.3 as the minimum cluster density for module detection.

Results show that three out of 2608 discovered modules were enriched (adjusted $p$-value < 0.05). Two modules (namely Modules 347 and 111) were found to have intra-QTL genes from qDTY12.1 interacting with intra-QTL genes found in qDTY2.3. Module 347 (adjusted $p$-value = 0.02821) has 55 genes and an interaction with qDTY2.3 intra-QTL gene LOC_OS02g52830 (lipase, putative, expressed) was reported. Module 111 (adjusted $p$-value = 0.02821) has 10 genes, of which an interaction with qDTY2.3 intra-QTL gene LOC_Os02g46480 (expressed protein) was reported. The interaction networks for these modules are shown in Figures 6 and 7.

From these analyses done using RicePilaf, a targeted set of novel candidates from the two reference genomes from subpopulations that are more closely related to the QTL donor varieties were identified, which were not reported at the time of the initial studies. Two additional candidate genes from interacting QTL qDTY2.3 were also identified. These additional candidates can be used in future studies to further understand the mechanism of yield-under-drought stress across various drought-tolerant rice varieties and drought-tolerance QTL interactions at the gene level.

### *Post-GWAS analysis of pre-harvest sprouting*

Pre-harvest sprouting (PHS) is a condition in which seeds lose dormancy and germinate prior to harvest, thus negatively affecting grain yield and quality [56]. A recent GWAS on PHS using a panel of 277 accessions representing temperate and tropical japonica and indica populations found the loci Chr01:1523,625-1770814 and Chr04: 4662701-4670717 to be significantly associated with this trait [12].

*Gene list and lift-over.* We lifted over the PHS loci to the indica IR64 genome since the PHS GWAS by Lee *et al.* [12] contains indica acces-

**Table 1.** Intra-QTL genes from the lift-over of QTL qDTY12.1 from Nipponbare that are unique to N22 genome

| N22 gene name | Description | UniProtKB/Swiss-Prot | OGI (Rice Gene Index ID) |
|---|---|---|---|
| ***In the same chromosome*** | | | |
| OsN22_12G011840 | | | OGI:12042120 |
| OsN22_12G011921 | | | OGI:12042590 |
| OsN22_12G012060 | | | OGI:12043500 |
| OsN22_12G012070 | | | OGI:12043530 |
| OsN22_12G012110 | | | OGI:12043640 |
| OsN22_12G012210 | | | OGI:06025660 |
| OsN22_12G012220 | | | OGI:12043990 |
| OsN22_12G012262 | | | OGI:12046180 |
| OsN22_12G012390 | No known annotation | No known mapping | OGI:12046090 |
| OsN22_12G012421 | | | OGI:12046130 |
| OsN22_12G012450 | | | OGI:12018740 |
| OsN22_12G012530 | | | OGI:12046710 |
| OsN22_12G012671 | | | OGI:12047920 |
| OsN22_12G012720 | | | OGI:12048080 |
| OsN22_12G013090 | | | OGI:03099600 |
| OsN22_12G013150 | | | OGI:12049060 |
| OsN22_12G013200 | | | OGI:12049140 |
| OsN22_12G013480 | Disease resistance protein RPS2 | Q42484 | OGI:12050040 |
| OsN22_12G012830 | Wall-associated receptor kinase 3 | Q9LMN8 | OGI:12048340 |
| OsN22_12G012870 | Receptor-like protein EIX2 | Q6JN46 | OGI:12048400 |
| ***In different chromosomes*** | | | |
| OsN22_01G004210 | No known annotation | No known mapping | OGI:01011120 |
| OsN22_06G029780 | | | OGI:06087140 |
| OsN22_09G011341 | | | OGI:09045920 |
| OsN22_06G017380 | | | OGI:06052670 |
| OsN22_10G003960 | (S)-N-methylcoclaurine 3'-hydroxylase isozyme 1 (Fragment) | Q9SP06 | OGI:10016160 |
| OsN22_09G011340 | Amino acid permease 4 | Q9FN04 | OGI:09045880 |
| OsN22_09G014850 | UDP-glycosyltransferase 88B1 | Q6VAA7 | OGI:09054580 |
| OsN22_02G022160 | Sugar transport protein MST1 | Q0JCR9 | OGI:02062760 |

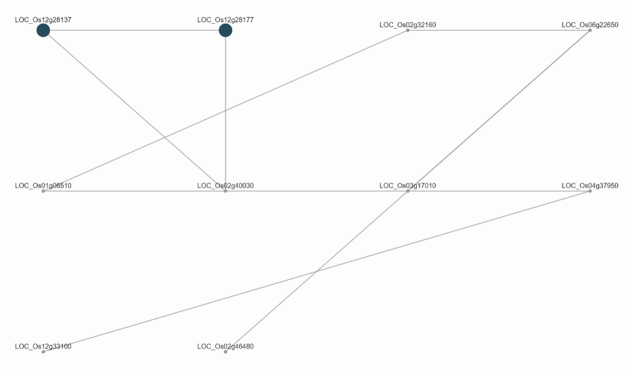


**Figure 7.** Co-expression network analysis using yield-under-drought QTL qDTY12.1 intra-QTL genes for Module 111 that interact with intra-QTL genes for QTL qDTY2.3 Module 111 includes intra-QTL gene LOC_Os02g46480. qDTY12.1 was reported to interact with qDTY2.3 by Dixit *et al.* [53]. The shaded nodes indicate genes that fall within the physical interval of qDTY12.1 (Chr12:15121175-1818433).

sions. We found 36 gene models overlapping the PHS loci in Nipponbare, of which 22 had orthologs in the corresponding IR64 intervals. Interestingly, of the genes unique to IR64, there were three whose Nipponbare orthologs were not contained in the original Nipponbare intervals (Table 3). These genes were not considered in the PHS GWAS by Lee *et al.* [12], demonstrating the benefit of lift-over. We included them in the 36 Nipponbare genes for further analysis.

*Co-expression network analysis.* Out of 2608 modules found by running ClusterONE on RiceNet v2 with the minimum cluster density set to 0.3, we found 39 modules that were enriched in the genes obtained in the previous step (adjusted $p$-value < 0.05). Without the three additional genes, there were 36 modules, further emphasizing the importance of RicePilaf's lift-over feature. These 36 modules provide a narrower list of candidate genes possibly involved in PHS that could be experimentally tested.

Among the top three enriched modules, namely Modules 690 (adjusted $p$-value = 0.001214), 2425 (adjusted $p$-value = 0.001214), and 901 (adjusted $p$-value = 0.001405), common enriched gene ontology terms include tryptophan biosynthetic process and serine-type carboxypeptidase (SCP) activity. Tryptophan has been reported to impact seed dormancy and PHS in wheat [57], and some SCPs and SCP-like proteins are known to be involved in the regulation of seed germination in rice and other crops [58, 59, 60]. In Module 690, the phytohormone jasmonic acid — which, along with its derivatives, is related to seed dormancy and germination [61, 62, 63] — appears as an enriched trait ontology term.

In Module 901, enriched gene ontology terms include the activities of β-glucosidase and β-amylase; certain genes belonging to these classes have been reported to be upregulated during pre-germination and early germination, presumably for their role in starch degradation [64], which corroborates with starch and sucrose metabolism also appearing as an enriched pathway. Another pathway of interest in this module is the biosynthesis of various secondary plant metabolites such as coumarin, the ability of which

**Table 2.** Intra-QTL genes from the lift-over of qDTY12.1 from Nipponbare that are unique to ARC 10497 genome

| ARC 10497 gene name | Description | UniProtKB/Swiss-Prot | OGI (Rice Gene Index ID) |
|---|---|---|---|
| *In the same chromosome* | | | |
| OsARC_12g0011921<br>OsARC_12g0011930<br>OsARC_12g0011950<br>OsARC_12g0012021<br>OsARC_12g0012030<br>OsARC_12g0012120<br>OsARC_12g0012170<br>OsARC_12g0012240<br>OsARC_12g0012250<br>OsARC_12g0012350<br>OsARC_12g0012500<br>OsARC_12g0012590<br>OsARC_12g0012730<br>OsARC_12g0012880<br>OsARC_12g0013120<br>OsARC_12g0013270<br>OsARC_12g0013350 | No known annotation | No known mapping | OGI:12038540<br>OGI:12042120<br>OGI:12042180<br>OGI:12042810<br>OGI:12042870<br>OGI:12043530<br>OGI:12043640<br>OGI:12043840<br>OGI:12014630<br>OGI:12045300<br>OGI:12046010<br>OGI:12018740<br>OGI:12046510<br>OGI:12048080<br>OGI:12048660<br>OGI:03099600<br>OGI:12049140 |
| OsARC_12g0013640 | Ent-sandaracopimara-8(14),15-diene synthase, chloroplastic | Q2QQJ5 | OGI:12050140 |
| OsARC_12g0012970 | Wall-associated receptor kinase 4 | Q9LMN6 | OGI:12048340 |
| OsARC_12g0013010 | Receptor-like protein EIX2 | Q6JN46 | OGI:12048400 |
| *In different chromosomes* | | | |
| OsARC_10g0006180<br>OsARC_06g0022101<br>OsARC_04g0000110<br>OsARC_10g0005060 | No known annotation | No known mapping | OGI:10026860<br>OGI:06069780<br>OGI:04000540<br>OGI:10020180 |

**Table 3.** Genes unique to IR64 in the pre-harvest sprouting GWAS loci

| Gene name | Description | UniProtKB/Swiss-Prot | Ortholog in Nipponbare |
|---|---|---|---|
| OsIR64_01g0001940 | Bowman-Birk type bran trypsin inhibitor | A2WK50 | LOC_Os01g03680 |
| OsIR64_01g0001950 | Putative cysteine-rich receptor-like protein kinase 33 | Q9LDN1 | LOC_Os01g03690 |
| OsIR64_01g0002290 | Mediator of RNA polymerase II transcription subunit 22a | Q9SA42 | LOC_Os01g04110 |
| OsIR64_01g0002000<br>OsIR64_01g0002030<br>OsIR64_01g0002040<br>OsIR64_01g0002190<br>OsIR64_01g0002200<br>OsIR64_01g0002240<br>OsIR64_01g0002260<br>OsIR64_01g0002270<br>OsIR64_01g0002280 | No known annotation | No known mapping | No known orthologs |

to inhibit abscisic acid catabolism has been used to block PHS and vivipary in rice [65].

Except for the starch and sucrose metabolism pathway, these aforementioned ontology terms and pathways were not reported in the PHS GWAS by Lee *et al.* [12], showing how RicePilaf's co-expression network analysis can provide further functional insights into rice GWAS loci. The genes in the discovered modules (as in Figure 8) can also be investigated experimentally for possible involvement in PHS and related biological processes.

To demonstrate the utility of RicePilaf providing multiple module detection algorithms in enriching post-GWAS analysis, we explored the enriched modules when the algorithm was set to FOX instead (with the weighted community clustering metric set to 0.05). Compared to when only the 36 gene models overlapping the PHS loci in Nipponbare were considered, the inclusion of the three Nipponbare orthologs from lift-over (Table 3) resulted in 10 additional enriched modules; in total, out of 4416 discovered modules, 34 modules were found to be enriched.

Among the top five enriched modules, Modules 1093 (adjusted $p$-value = 0.02398) and 1331 (adjusted $p$-value = 0.02398) in particular are enriched in germination- and growth-associated ontology terms related to the activity of key phytohormones (e.g., regulation of auxin biosynthetic process, auxin homeostasis, and gibberellic acid homeostasis), plant development (e.g., shoot system development and lateral root development), and metabolic activities (e.g., sucrose metabolic process and regulation of starch biosynthetic process). Plant embryo stage and seedling development stage also appear as enriched plant ontology terms in Module 1331.

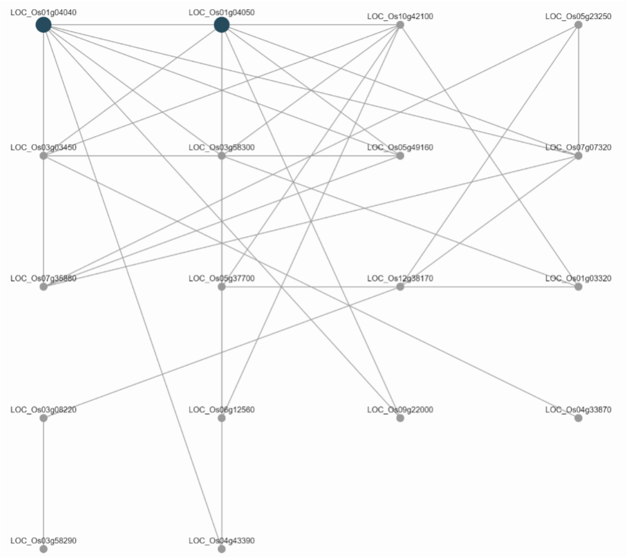


**Figure 8.** Co-expression network analysis of the loci Chr01:1523,625-1770814 and Chr04:4662701-4670717, known to be significantly associated with pre-harvest sprouting [12]. The graph is a module (Module 690) that is enriched in ontology terms and pathways related to seed germination, dormancy, and vivipary, such as activities of β-glucosidase, β-amylase, starch and sucrose metabolism, and biosynthesis of coumarin. The shaded nodes indicate genes that fall within the specified loci.

*Enrichment in transcription factor binding sites.* The top transcription factors whose binding sites significantly overlap with the PHS loci were CAMTA, FAR1, and ERF (each with an adjusted $p$-value of 0.1). These transcription factor families have been reported to be involved in biotic and abiotic stress response [66, 67].

## Limitations

RicePilaf integrates several existing tools and methods and depends on currently available datasets (Table 4), and thus carries the limitations inherent in those tools and data. For example, genomes and gene models are only available for the reference genomes currently available at the time of writing; however, we intend to update genomes periodically on the public site. Data on regulatory and co-expression networks in rice depend on the currently available data from a limited number of tissues and conditions.

RicePilaf currently also lacks the means to incorporate tissue-specific gene expression information to narrow down the list of candidate genes, since currently available expression data have a limited range of tissues or use older technology that is not straightforward to integrate (e.g. [68, 69]). Similarly, epigenomic data such as chromatin accessibility and histone modification marks, which are known to be condition- and tissue-specific, are currently available for a very limited variety of tissues and come from only a few samples. In the lift-over functionality, there may be limitations related to possible multiplicity of alignment (in case of large segmental duplications). Currently, we force LAST to output only one-to-one alignments; thus, any homologous regions due to duplications are not possible to interrogate.

## Outlook

### Handling database updates

RicePilaf integrates information from multiple databases that are bound to see upgrades in the future. Additionally, in order for RicePilaf to respond quickly to user queries, we preprocess raw data from these databases to precompute information such as alignments, module detection, identification of enriched modules, ontology and pathway enrichment analysis, and annotations of PubMed abstracts. We incorporated the scripts for downloading and preprocessing into a Snakemake pipeline [70]. These scripts, along with all the necessary dependencies, are bundled into a Docker image (separate from the image for running the app), which can be downloaded from the code repository.

### Adding new features

RicePilaf follows a modular and extensible design that allows for easy addition and updating of features in the future. One key feature of immediate interest is collecting additional information from remote RESTful application programming interfaces (APIs). Databases that provide API access include UniProt [71], Gramene [72], and AgroLD [73]. Another key feature is including complementary data retrieval APIs like PyRice [28] to expand and facilitate a broader search of information.

## Conclusion

RicePilaf enables rice breeders and scientists to quickly cross-reference GWAS/QTL analysis results with a variety of rice databases. There have been several publications that identify potential QTLs and GWAS regions that remain poorly characterized for their specific mechanisms, and the overarching philosophy that drives this software development effort is the desire to solve this big unknown. This software platform is intended as a tool in order to understand many other QTLs' functionality and figure out ways of dissecting the mechanistic granularities. Otherwise, we will just be building layers over layers without getting to the core. As an example, there are at least three other QTLs identified at the International Rice Research Institute now that operate as multigene QTLs. Hence, having an analysis pipeline for dissecting such regions for critical genes will add value as we and others discover more multigene QTLs in the future, especially in rice, mainly due to its compact genome. RicePilaf is easy to install, as it simply requires downloading a Docker image, along with the preprocessed dataset, and spinning up the container. It is also easy to use as it runs on a browser providing a user-friendly interface and a set of interactive web reports.

## Methods

### Lift-over

RicePilaf can translate GWAS/QTL regions on Nipponbare to genomic intervals of other accessions representing major Asian rice populations. Currently, the choice of genome as lift-over target includes *(i)* tropical Japonica Azucena, *(ii)* sub-tropical Japonica CHAO MEO, *(iii)* circum-Aus N22, *(iv)* indica IR64, *(v)* indica MH63, and *(vi)* circum-Basmati ARC 10497.

The lift-over from Nipponbare to a target genome is performed as follows. Pairwise whole-genome sequence alignment between Nipponbare and the target genome is precomputed using LAST as described in [75]. LAST produces a set of one-to-one local alignments, i.e., a base pair in Nipponbare aligns to at most one base pair in the target and vice versa. Additionally, there is no constraint for the alignments to be co-linear, which allows for capturing complex inter or intrachromosomal genome rearrangements. These precomputed alignments map Nipponbare genomic intervals to orthologous regions in the target. The set of gene models overlapping the target intervals is obtained from the genome annotations provided by Rice Gene Index [24]. The same project also provides orthologous gene groups, which can be used to compare GWAS/QTL gene sets across different accessions.

**Table 4.** Summary of datasets used

| Dataset type | Project, URL | Publication |
|---|---|---|
| Genome sequences, annotation, gene descriptions, and orthology maps of rice varieties | Rice Gene Index https://riceome.hzau.edu.cn/ | Yu *et al.* [24] |
| Co-expression network | RiceNet v2 https://www.inetbio.org/ricenet/ | Lee *et al.* [41] |
| Co-expression network | Rice Combined Mutual Ranked Network (RCRN) https://doi.org/10.5061/dryad.zgmsbcc69 | Zhao *et al.* [42] |
| Gene ontology annotations | Rice Annotation Project Database (RAP-DB) https://rapdb.dna.affrc.go.jp/ | Sakai *et al.* [74] |
| Gene ontology annotations | agriGO v2.0 http://systemsbiology.cau.edu.cn/agriGOv2/ | Tian *et al.* [43] |
| Gene, plant, and trait ontology annotations | Oryzabase https://shigen.nig.ac.jp/rice/oryzabase/locale/change?lang=en | Kurata & Yamazaki [45] |
| Pathway maps | Kyoto Encyclopedia of Genes and Genomes (KEGG) https://www.genome.jp/kegg/ | Kanehisa & Goto [44] |
| TF binding sites | PlantRegMap http://plantregmap.gao-lab.org/ | Tian *et al.* [50] |
| QTL from published literature | QTARO http://qtaro.abr.affrc.go.jp/ accessed 2016-06 | Yonemaru *et al.* [29] |
| Open chromatin | RiceENCODE http://glab.hzau.edu.cn/RiceENCODE/ | Xie *et al.* [52] |

## Co-expression network analysis

RicePilaf integrates co-expression information in three stages: detection of modules (also known as communities or clusters), identification of modules enriched in the GWAS/QTL genes, and functional characterization of these modules by ontology and pathway enrichment analysis. We describe these steps in detail below.

### *Module/community detection*

First, RicePilaf identifies modules of genes given a co-expression network; users can select either RiceNet v2 [41] or the Rice Combined Mutual Ranked Network (RCRN) [42]. For RiceNet v2, the co-expression network used is the component network derived from the co-expression of *Oryza sativa* genes across microarray experiments. For RCRN, the integrated network is used.

Since genes can possibly be involved in multiple biological functions or processes [76], the supported module detection algorithms allow for overlapping modules, i.e., a given gene may belong to multiple modules. To this end, RicePilaf provides a selection of four algorithms: *(i)* ClusterONE [77], *(ii)* COACH [78], *(iii)* DEMON [79], and *(iv)* FOX [80].

### *Identification of enriched modules*

From among the detected gene modules, RicePilaf performs over-representation analysis to identify which modules are statistically enriched in view of the co-expression network and the GWAS/QTL-implicated genes. To this end, a $2{\times}2$ contingency table is constructed, with all the genes across the detected modules comprising the background gene set. The columns count the number of genes implicated by GWAS/QTL (versus those that are not implicated), and the rows count the number of genes present in the module being tested (versus those that are not present). A one-tailed Fisher's exact test is then applied, followed by multiple-testing correction via the Benjamini-Hochberg method [81]. A module is considered enriched if its adjusted $p$-value is less than 0.05.

### *Functional characterization via ontology and pathway enrichment analysis*

The likely biological functions of the enriched modules are inferred by performing enrichment analysis across several ontology and pathway databases. For the ontology enrichment analysis, RicePilaf displays results for three sets of ontologies: *(i)* gene ontology, *(ii)* trait ontology, and *(iii)* plant ontology. Gene ontology annotations — which cover cellular components, molecular functions, and biological processes — are aggregated from the Rice Annotation Project Database (RAP-DB) [74], agriGO v2.0 [43], and Oryzabase [45]. Trait and plant ontology annotations — which focus on phenotypic attributes — are obtained from Oryzabase [45].

For identifying enriched pathways, RicePilaf supports both over-representation analysis via clusterProfiler [82] and topology-based analysis via Pathway-Express [83] and Signaling Pathway Impact Analysis (SPIA) [84]. Pathway maps are obtained from the Kyoto Encyclopedia of Genes and Genomes (KEGG) [44]; accessions are mapped to KEGG identifiers using the R package `riceidconverter` [85] and mapping tables from RAP-DB [74]. An ontology term or pathway is considered enriched if its adjusted $p$-value after Benjamini-Hochberg correction [81] is less than 0.05.

## Enrichment of regulatory features

RicePilaf determines if the input GWAS/QTL intervals are enriched for binding sites of transcription factors (TFs). This is done by first computing overlaps between GWAS/QTL genomic intervals and predicted binding sites of a TF using Pybedtools [86, 87]. For TFs that have a non-empty intersection, the statistical significance of the overlap is computed using MCDP2 [88]; and multiple testing across multiple TFs is accounted for by Benjamini-Hochberg correction of the significance values [81]. Binding site information of almost 250 TFs is obtained from PlantRegMap [50]. For each TF, PlantRegMap provides several sets of predicted binding sites depending on how the prediction was performed — (i) simple motif scanning using

FIMO [89] or (ii) motif scanning paired with conserved element information [50] or (iii) using the FunTFBS method [50] — and what target sequence was used — whole genome versus promoter region defined as −500/+100 bp of the transcription start site. RicePilaf exposes these choices to the user.

### Text mining

Around 17,000 scientific abstracts were retrieved from PubMed by using a curated list of PubMed identifiers provided by the Oryzabase database [45]. This list provides manually checked PubMed entries related to rice genomics. A natural language processing pipeline was written using Python to extract named entities from these abstracts. This pipeline combines the HunFLAIR named entity recognition tagger [90] with spaCy [91], Natural Language Toolkit (NLTK) [92], and other libraries. It identifies four types of named entity annotations: gene names (e.g., "OsMAPK2" or "MOC1"), species (e.g., "Oryza sativa" or "Magnaporthe oryzae"), chemicals (e.g., "gibberellic acid" or "nitrogen") and disease or phenotype (e.g., "blast disease" or "sheath blight disease"). In total, 351,003 annotations of 63,591 distinct named entities were identified from these PubMed abstracts and titles.

RicePilaf performs quick searches on these annotations to retrieve gene names associated with diseases, phenotypes, chemicals, or species. The retrieved genes can be added to the co-expression network and regulatory enrichment analyses, thus further enriching the post-GWAS analysis.

### A summary of datasets used

A summary of the datasets used in RicePilaf is presented in Table 4.

## Availability of source code and requirements

- Project name: RicePilaf
- Project home page: The source code and URLs to publicly accessible running instances of RicePilaf are available at https://github.com/bioinfodlsu/rice-pilaf.
- Operating system(s): Platform-independent
- Programming language: Python Dash and Flask, R, Snakemake
- Other requirements: web browser, Docker (for installing locally or deploying to a server)
- License: MIT License
- SciCrunch RRID: SCR_024945
- bio.tools URL: https://bio.tools/ricepilaf

## Data availability

The URL to the dataset required for running the app can be found in the project's repository. This dataset has been generated from publicly available datasets described in Table 4 using the Snakemake pipeline available in the project's code repository.

Other supporting materials are available in Software Heritage Archive [93].

## Declarations

### List of abbreviations

GWAS: Genome-wide association study; QTL: Quantitative trait loci; SNP: Single-nucleotide polymorphism; SSR: Simple sequence repeat; LD: Linkage disequilibrium; TF: Transcription factor; SCP: Serine-type carboxypeptidase; PHS: Pre-harvest sprouting; API: Application programming interface; RGI: Rice Gene Index; RCRN: Rice Combined Mutual Ranked Network; RAP-DB: Rice Annotation Project Database; KEGG: Kyoto Encyclopedia of Genes and Genomes; NLTK: Natural Language Toolkit

### Ethical Approval (optional)

Not applicable.

### Consent for publication

Not applicable.

### Competing Interests

The author(s) declare that they have no competing interests.

### Funding

This research was funded by the Rural Development Administration (RDA) of South Korea as an RDA-IRRI cooperative project (Grant number PJ016405, PI: Dr. Ji-Ung Jeong). The funding body had no role in collection, analysis, or interpretation of the data.

### Authors' Contributions

AMSS, MEMG, and PCLO: Methodology, Software, Writing - Original Draft. PL: Methodology, Software, Writing - Original Draft. DC, RPM, KLM: Conceptualization, Writing - Review and Editing. JL, KLM: Funding Acquisition, Project Administration.

## Acknowledgements

We thank Jianwei Zhang and Zhichao Yu for providing orthology maps from the Rice Gene Index. We thank Elyssia Barrie Ong for advice on the app's front-end design. We thank Jeffrey Detras and Richard Pasco for assistance in setting up a demo RicePilaf instance at the Philippine Government Department of Science and Technology – Advanced Science and Technology Institute (DOST-ASTI) COARE-Science Cloud resources, and DOST-ASTI for hosting the instance. We acknowledge the ISO 9001 certified IRD i-Trop HPC (South Green Platform) at IRD montpellier for providing HPC resources that have contributed to the research results reported within this paper. URL: https://bioinfo.ird.fr http://www.southgreen.fr.

## References

1. Wing RA, Purugganan MD, Zhang Q. The rice genome revolution: from an ancient grain to Green Super Rice. Nat Rev Genet 2018 Aug;19(8):505–517.
2. Jeong BY, Lee Y, Kwon Y, Kim JH, Ham TH, Kwon SW, et al. Genome-Wide Association Study Reveals the Genetic Basis of Chilling Tolerance in Rice at the Reproductive Stage. Plants 2021 Aug;10(8).
3. Ravikiran KT, Gopala Krishnan S, Abhijith KP, Bollinedi H, Nagarajan M, Vinod KK, et al. Genome-Wide Association Mapping Reveals Novel Putative Gene Candidates Governing Reproductive Stage Heat Stress Tolerance in Rice. Front Genet 2022 May;13:876522.
4. Li P, Jiang J, Zhang G, Miao S, Lu J, Qian Y, et al. Integrating GWAS and transcriptomics to identify candidate genes conferring heat tolerance in rice. Front Plant Sci 2022;13:1102938.
5. Tnani H, Chebotarov D, Thapa R, Ignacio JCI, Israel WK, Quilloy FA, et al. Enriched-GWAS and Transcriptome Analysis to Re-

fine and Characterize a Major QTL for Anaerobic Germination Tolerance in Rice. Int J Mol Sci 2021 Apr;22(9).
6. Kong W, Li S, Zhang C, Qiang Y, Li Y. Combination of quantitative trait locus (QTL) mapping and transcriptome analysis reveals submerged germination QTLs and candidate genes controlling coleoptile length in rice. Food Energy Secur 2022 Feb;11(1).
7. Volante A, Tondelli A, Desiderio F, Abbruscato P, Menin B, Biselli C, et al. Genome wide association studies for japonica rice resistance to blast in field and controlled conditions. Rice 2020 Oct;13(1):71.
8. Zhao J, Yang W, Zhang S, Yang T, Liu Q, Dong J, et al. Genome-wide association study and candidate gene analysis of rice cadmium accumulation in grain in a diverse rice collection. Rice 2018 Nov;11(1):61.
9. Kong W, Deng X, Yang J, Zhang C, Sun T, Ji W, et al. High-resolution bin-based linkage mapping uncovers the genetic architecture and heterosis-related loci of plant height in indica-japonica derived populations. Plant J 2022 May;110(3):814–827.
10. Kong W, Deng X, Liao Z, Wang Y, Zhou M, Wang Z, et al. De novo assembly of two chromosome-level rice genomes and bin-based QTL mapping reveal genetic diversity of grain weight trait in rice. Front Plant Sci 2022 Aug;13:995634.
11. Wang A, Jiang Y, Shu X, Zha Z, Yin D, Liu Y, et al. Genome-wide association study-based identification genes influencing agronomic traits in rice (Oryza sativa L.). Genomics 2021 May;113(3):1396–1406.
12. Lee JS, Chebotarov D, McNally KL, Pede V, Setiyono TD, Raquid R, et al. Novel Sources of Pre-Harvest Sprouting Resistance for Japonica Rice Improvement. Plants 2021 Aug;10(8).
13. Lee JS, Velasco-Punzalan M, Pacleb M, Valdez R, Kretzschmar T, McNally KL, et al. Variation in seed longevity among diverse Indica rice varieties. Ann Bot 2019 Oct;124(3):447–460.
14. Wang W, Mauleon R, Hu Z, Chebotarov D, Tai S, Wu Z, et al. Genomic variation in 3,010 diverse accessions of Asian cultivated rice. Nature 2018 May;557(7703):43–49.
15. Mather KA, Caicedo AL, Polato NR, Olsen KM, McCouch S, Purugganan MD. The extent of linkage disequilibrium in rice (Oryza sativa L.). Genetics 2007 Dec;177(4):2223–2232.
16. Zhang P, Zhong K, Shahid MQ, Tong H. Association Analysis in Rice: From Application to Utilization. Front Plant Sci 2016 Aug;7:1202.
17. McCouch SR, Wright MH, Tung CW, Maron LG, McNally KL, Fitzgerald M, et al. Open access resources for genome-wide association mapping in rice. Nat Commun 2016 Feb;7:10532.
18. Morales KY, Singh N, Perez FA, Ignacio JC, Thapa R, Arbelaez JD, et al. An improved 7K SNP array, the C7AIR, provides a wealth of validated SNP markers for rice breeding and genetics studies. PLoS One 2020 May;15(5):e0232479.
19. Peat G, Jones W, Nuhn M, Marugán JC, Newell W, Dunham I, et al. The open targets post-GWAS analysis pipeline. Bioinformatics 2020 May;36(9):2936–2937.
20. Schaefer RJ, Michno JM, Jeffers J, Hoekenga O, Dilkes B, Baxter I, et al. Integrating Coexpression Networks with GWAS to Prioritize Causal Genes in Maize. Plant Cell 2018 Dec;30(12):2922–2942.
21. Juanillas V, Dereeper A, Beaume N, Droc G, Dizon J, Mendoza JR, et al. Rice Galaxy: an open resource for plant science. Gigascience 2019 May;8(5).
22. CGIAR Research Program on Rice, CropGalaxy - a unified analysis platform for multiple crops.; 2020. http://cropgalaxy.excellenceinbreeding.org/.
23. Purcell S, Neale B, Todd-Brown K, Thomas L, Ferreira MAR, Bender D, et al. PLINK: a tool set for whole-genome association and population-based linkage analyses. Am J Hum Genet 2007 Sep;81(3):559–575.
24. Yu Z, Chen Y, Zhou Y, Zhang Y, Li M, Ouyang Y, et al. Rice Gene Index: A comprehensive pan-genome database for comparative and functional genomics of Asian rice. Mol Plant 2023 Mar;.
25. UniProt Consortium. UniProt: the Universal Protein Knowledgebase in 2023. Nucleic Acids Res 2023 Jan;51(D1):D523–D531.
26. Paysan-Lafosse T, Blum M, Chuguransky S, Grego T, Pinto BL, Salazar GA, et al. InterPro in 2022. Nucleic Acids Res 2023 Jan;51(D1):D418–D427.
27. Mistry J, Chuguransky S, Williams L, Qureshi M, Salazar GA, Sonnhammer ELL, et al. Pfam: The protein families database in 2021. Nucleic Acids Res 2021 Jan;49(D1):D412–D419.
28. Do Q, Bich Hai H, Larmande P. PyRice: a Python package for querying Oryza sativa databases. Bioinformatics 2020 07;37(7):1037–1038.
29. Yonemaru JI, Yamamoto T, Fukuoka S, Uga Y, Hori K, Yano M. Q-TARO: QTL Annotation Rice Online Database. Rice 2010 Jun;3(2):194–203.
30. Zhou Y, Chebotarov D, Kudrna D, Llaca V, Lee S, Rajasekar S, et al. A platinum standard pan-genome resource that represents the population structure of Asian rice. Scientific Data 2020 Apr;7(1):1–11.
31. Stein JC, Yu Y, Copetti D, Zwickl DJ, Zhang L, Zhang C, et al. Genomes of 13 domesticated and wild rice relatives highlight genetic conservation, turnover and innovation across the genus Oryza. Nature Genetics 2018 Feb;50(2):285–296.
32. Song JM, Xie WZ, Wang S, Guo YX, Koo DH, Kudrna D, et al. Two gap-free reference genomes and a global view of the centromere architecture in rice. Molecular Plant 2021;14(10):1757–1767.
33. Fuentes RR, Chebotarov D, Duitama J, Smith S, De la Hoz JF, Mohiyuddin M, et al. Structural variants in 3000 rice genomes. Genome Research 2019 May;29(5):870–880.
34. Zhou Y, Yu Z, Chebotarov D, Chougule K, Lu Z, Rivera LF, et al. Pan-genome inversion index reveals evolutionary insights into the subpopulation structure of Asian rice. Nature Communications 2023 Mar;14(1):1567. Number: 1 Publisher: Nature Publishing Group.
35. Xu K, Xu X, Fukao T, Canlas P, Maghirang-Rodriguez R, Heuer S, et al. Sub1A is an ethylene-response-factor-like gene that confers submergence tolerance to rice. Nature 2006 Aug;442(7103):705–708. Number: 7103 Publisher: Nature Publishing Group.
36. Rao X, Dixon RA. Co-expression networks for plant biology: why and how. Acta Biochimica et Biophysica Sinica 2019 08;51(10):981–988.
37. Zhang C, Lee S, Mardinoglu A, Hua Q. Investigating the Combinatory Effects of Biological Networks on Gene Co-expression. Front Physiol 2016 May;7:160.
38. Ficklin SP, Feltus FA. Gene Coexpression Network Alignment and Conservation of Gene Modules between Two Grass Species: Maize and Rice. Plant Physiology 2011 05;156(3):1244–1256.
39. Almeida-Silva F, Venancio TM. Integration of genome-wide association studies and gene coexpression networks unveils promising soybean resistance genes against five common fungal pathogens. Sci Rep 2021 Dec;11(1):24453.
40. Calabrese GM, Mesner LD, Stains JP, Tommasini SM, Horowitz MC, Rosen CJ, et al. Integrating GWAS and Co-expression Network Data Identifies Bone Mineral Density Genes SPTBN1 and MARK3 and an Osteoblast Functional Module. Cell Syst 2017 Jan;4(1):46–59.e4.
41. Lee T, Oh T, Yang S, Shin J, Hwang S, Kim CY, et al. RiceNet v2: an improved network prioritization server for rice genes. Nucleic Acids Research 2015 Mar;43(W1):W122–W127.
42. Zhao K, Lin F, Romero-Gamboa SP, Saha P, Goh HJ, An G, et al. Rice Genome-Scale Network Integration Reveals Transcriptional Regulators of Grass Cell Wall Synthesis. Front Plant Sci 2019 Oct;10:1275.
43. Tian T, Liu Y, Yan H, You Q, Yi X, Du Z, et al. agriGO v2.0: a GO

analysis toolkit for the agricultural community, 2017 update. Nucleic Acids Research 2017 Jul;45(W1):W122–W129.
44. Kanehisa M, Goto S. KEGG: Kyoto encyclopedia of genes and genomes. Nucleic Acids Research 2000 Jan;28(1):27–30.
45. Kurata N, Yamazaki Y. Oryzabase. An Integrated Biological and Genome Information Database for Rice. Plant Physiol 2006 Jan;140(1):12.
46. 3,000 rice genomes project. The 3,000 rice genomes project. Gigascience 2014 May;3:7.
47. Deplancke B, Alpern D, Gardeux V. The Genetics of Transcription Factor DNA Binding Variation. Cell 2016 Jul;166(3):538–554.
48. Yao Q, Ferragina P, Reshef Y, Lettre G, Bauer DE, Pinello L. Motif-Raptor: a cell type-specific and transcription factor centric approach for post-GWAS prioritization of causal regulators. Bioinformatics 2021 Aug;37(15):2103–2111.
49. Schmidt EM, Zhang J, Zhou W, Chen J, Mohlke KL, Chen YE, et al. GREGOR: evaluating global enrichment of trait-associated variants in epigenomic features using a systematic, data-driven approach. Bioinformatics 2015 Aug;31(16):2601–2606.
50. Tian F, Yang DC, Meng YQ, Jin J, Gao G. PlantRegMap: charting functional regulatory maps in plants. Nucleic Acids Research 2020 Jan;48(D1):D1104–D1113.
51. Robinson JT, Thorvaldsdottir H, Turner D, Mesirov JP. igv.js: an embeddable JavaScript implementation of the Integrative Genomics Viewer (IGV). Bioinformatics 2023 Jan;39(1).
52. Xie L, Liu M, Zhao L, Cao K, Wang P, Xu W, et al. RiceENCODE: A comprehensive epigenomic database as a rice Encyclopedia of DNA Elements. Mol Plant 2021 Oct;14(10):1604–1606.
53. Dixit S, Mallikarjuna Swamy BP, Vikram P, Bernier J, Sta Cruz MT, Amante M, et al. Increased drought tolerance and wider adaptability of qDTY 12.1 conferred by its interaction with qDTY 2.3 and qDTY 3.2. Mol Breed 2012 Dec;30(4):1767–1779.
54. Dixit S, Kumar Biswal A, Min A, Henry A, Oane RH, Raorane ML, et al. Action of multiple intra-QTL genes concerted around a co-localized transcription factor underpins a large effect QTL. Sci Rep 2015 Oct;5:15183.
55. Mishra KK, Vikram P, Yadaw RB, Swamy BPM, Dixit S, Cruz MTS, et al. qDTY12.1: a locus with a consistent effect on grain yield under drought in rice. BMC Genet 2013 Feb;14:12.
56. Lee CM, Park HS, Baek MK, Jeong OY, Seo J, Kim SM. QTL mapping and improvement of pre-harvest sprouting resistance using japonica weedy rice. Front Plant Sci 2023 Jun;14:1194058.
57. Ramaih S, Guedira M, Paulsen GM. Relationship of indoleacetic acid and tryptophan to dormancy and preharvest sprouting of wheat. Funct Plant Biol 2003 Oct;30(9):939–945.
58. Li Z, Tang L, Qiu J, Zhang W, Wang Y, Tong X, et al. Serine carboxypeptidase 46 Regulates Grain Filling and Seed Germination in Rice (Oryza sativa L.). PLoS One 2016 Jul;11(7):e0159737.
59. Cercós M, Urbez C, Carbonell J. A serine carboxypeptidase gene (PsCP), expressed in early steps of reproductive and vegetative development in Pisum sativum, is induced by gibberellins. Plant Mol Biol 2003 Jan;51(2):165–174.
60. Jiang P, Gao J, Mu J, Duan L, Gu Y, Han S, et al. Interaction between serine carboxypeptidase-like protein TtGS5 and Annexin D1 in developing seeds of Triticum timopheevi. J Appl Genet 2020 Jan;61(2):151–162.
61. Wang Y, Hou Y, Qiu J, Wang H, Wang S, Tang L, et al. Abscisic acid promotes jasmonic acid biosynthesis via a 'SAPK10-bZIP72-AOC' pathway to synergistically inhibit seed germination in rice (Oryza sativa). New Phytol 2020 Jul;228(4):1336–1353.
62. Sohn SI, Pandian S, Kumar TS, Zoclanclounon YAB, Muthuramalingam P, Shilpha J, et al. Seed Dormancy and Pre-Harvest Sprouting in Rice-An Updated Overview. Int J Mol Sci 2021 Oct;22(21).
63. Trang Nguyen H, Thi Mai To H, Lebrun M, Bellafiore S, Champion A. Jasmonates-the Master Regulator of Rice Development, Adaptation and Defense. Plants (Basel) 2019 Sep;8(9).
64. Li H, Li X, Wang G, Zhang J, Wang G. Analysis of gene expression in early seed germination of rice: landscape and genetic regulation. BMC Plant Biology 2022 Feb;22(1):70.
65. Chen BX, Peng YX, Gao JD, Zhang Q, Liu QJ, Fu H, et al. Coumarin-Induced Delay of Rice Seed Germination Is Mediated by Suppression of Abscisic Acid Catabolism and Reactive Oxygen Species Production. Front Plant Sci 2019 Jun;10:828.
66. Nakano T, Suzuki K, Fujimura T, Shinshi H. Genome-wide analysis of the ERF gene family in Arabidopsis and rice. Plant Physiol 2006 Jan;140(2):411–432.
67. Gain H, Nandi D, Kumari D, Das A, Dasgupta SB, Banerjee J. Genome-wide identification of CAMTA gene family members in rice (Oryza sativa L.) and in silico study on their versatility in respect to gene expression and promoter structure. Funct Integr Genomics 2022 Feb;22(2):193–214.
68. Sato Y, Takehisa H, Kamatsuki K, Minami H, Namiki N, Ikawa H, et al. RiceXPro version 3.0: expanding the informatics resource for rice transcriptome. Nucleic Acids Res 2013 Jan;41(Database issue):D1206–13.
69. Kawahara Y, Oono Y, Wakimoto H, Ogata J, Kanamori H, Sasaki H, et al. TENOR: Database for Comprehensive mRNA-Seq Experiments in Rice. Plant Cell Physiol 2016 Jan;57(1):e7.
70. Mölder F, Jablonski K, Letcher B, Hall M, Tomkins-Tinch C, Sochat V, et al. Sustainable data analysis with Snakemake. F1000Research 2021;10(33).
71. The UniProt Consortium. UniProt: the Universal Protein Knowledgebase in 2023. Nucleic Acids Research 2023 Jan;51(D1):D523–D531.
72. Tello-Ruiz MK, Naithani S, Gupta P, Olson A, Wei S, Preece J, et al. Gramene 2021: harnessing the power of comparative genomics and pathways for plant research. Nucleic Acids Research 2021 Jan;49(D1):D1452–D1463.
73. Venkatesan A, Tagny Ngompe G, Hassouni NE, Chentli I, Guignon V, Jonquet C, et al. Agronomic Linked Data (AgroLD): A knowledge-based system to enable integrative biology in agronomy. PLOS ONE 2018;13(11):1–17.
74. Sakai H, Lee SS, Tanaka T, Numa H, Kim J, Kawahara Y, et al. Rice Annotation Project Database (RAP-DB): an integrative and interactive database for rice genomics. Plant Cell Physiol 2013 Jan;54(2):e6.
75. Frith MC, Kawaguchi R. Split-alignment of genomes finds orthologies more accurately. Genome Biol 2015 May;16(1):106.
76. Pritykin Y, Ghersi D, Singh M. Genome-Wide Detection and Analysis of Multifunctional Genes. PLoS Comput Biol 2015 Oct;11(10):e1004467.
77. Nepusz T, Yu H, Paccanaro A. Detecting overlapping protein complexes in protein-protein interaction networks. Nature Methods 2012 May;9(5):471–472.
78. Wu M, Li X, Kwoh CK, Ng SK. A core-attachment based method to detect protein complexes in PPI networks. BMC Bioinformatics 2009 Jun;10:169.
79. Coscia M, Rossetti G, Giannotti F, Pedreschi D. DEMON: A Local-First Discovery Method for Overlapping Communities. KDD '12, New York, NY, USA: Association for Computing Machinery; 2012. p. 615–623.
80. Lyu T, Bing L, Zhang Z, Zhang Y. FOX: Fast Overlapping Community Detection Algorithm in Big Weighted Networks. Trans Soc Comput 2020 aug;3(3).
81. Benjamini Y, Hochberg Y. Controlling the False Discovery Rate: A Practical and Powerful Approach to Multiple Testing. Journal of the Royal Statistical Society: Series B (Methodological) 1995;57(1):289–300.
82. Wu T, Hu E, Xu S, Chen M, Guo P, Dai Z, et al. clusterProfiler 4.0: A universal enrichment tool for interpreting omics data. The Innovation 2021;2(3):100141.
83. Draghici S, Khatri P, Tarca AL, Amin K, Done A, Voichita C,

et al. A systems biology approach for pathway level analysis. Genome Res 2007 Sep;17(10):1537–1545.
84. Tarca AL, Draghici S, Khatri P, Hassan SS, Mittal P, Kim Js, et al. A novel signaling pathway impact analysis. Bioinformatics 2008 11;25(1):75–82.
85. Li X, riceidconverter: Convert Biological ID from RAP or MSU to SYMBOL for Oryza Sativa; 2020. https://cran.r-project.org/web/packages/riceidconverter/index.html.
86. Dale RK, Pedersen BS, Quinlan AR. Pybedtools: a flexible Python library for manipulating genomic datasets and annotations. Bioinformatics 2011 Dec;27(24):3423–3424.
87. Quinlan AR, Hall IM. BEDTools: a flexible suite of utilities for comparing genomic features. Bioinformatics 2010 Mar;26(6):841–842.
88. Gafurov A, Brejová B, Medvedev P. Markov chains improve the significance computation of overlapping genome annotations. Bioinformatics 2022 Jun;38(Suppl 1):i203–i211.
89. Grant CE, Bailey TL. FIMO: scanning for occurrences of a given motif. Bioinformatics 2011 Feb;27(7):1017–1018.
90. Weber L, Sänger M, Münchmeyer J, Habibi M, Leser U, Akbik A. HunFlair: an easy-to-use tool for state-of-the-art biomedical named entity recognition. Bioinformatics 2021 01;37(17):2792–2794.
91. Honnibal M, Montani I. spaCy 2: Natural language understanding with Bloom embeddings, convolutional neural networks and incremental parsing; 2017.
92. Bird S, Loper E. NLTK: The Natural Language Toolkit. In: Proceedings of the ACL Interactive Poster and Demonstration Sessions Barcelona, Spain: Association for Computational Linguistics; 2004. p. 214–217.
93. Shrestha AMS, Gonzales MEM, Ong PCL, Larmande P, Lee HS, Jeung JU, et al., RicePilaf (Version 0.1.2). [Computer software], Software Heritage; 2024. swh:1:dir:0eba216d37b5279256314e7d5412190d520ca4a1; origin=https://github.com/bioinfodlsu/rice-pilaf; visit=swh:1:snp:603c49ff669503658d92810346116e6dd9a8b79b; anchor=swh:1:rev:9c5d9c248336cc7f376f5f38fb757ed2826b976e.
