# Supplementary material for: RicePilaf: a post-GWAS/QTL dashboard to integrate pangenomic, coexpression, regulatory, epigenomic, ontology, pathway, and text-mining information to provide functional insights into rice QTLs and GWAS loci
Source: Gigascience. 2024 Jun 4;13:giae013. doi: 10.1093/gigascience/giae013 (PMC11148593; doi:10.1093/gigascience/giae013)
Supplement: giae013_Response_to_Reviewer_Comments_Original_Submission [file giae013_response_to_reviewer_comments_original_submission.pdf]

Author's Response To Reviewer Comments

Dear Mr. Edmunds,

We are pleased to submit our revised Technical note entitled “RicePilaf: a post-GWAS/QTL dashboard to integrate pangenomic, co-expression, regulatory, epigenomic, ontology, pathway, and text-mining information to provide functional insights into rice QTLs and GWAS loci” for your consideration. We would like to thank the editors and reviewers for their constructive comments. Based on their comments, we have implemented a major feature update of the software and updated the manuscript to reflect the changes. Changes in the manuscript are marked in red. With these, we have addressed almost all the issues raised, the only exception being a suggestion from Reviewer 1 to include the gene expression database RiceXPro which was not possible due to missing mapping information between microarray spots and RGAP gene IDs. A detailed point-by-point response follows.

Thank you for considering our submission.

Sincerely,
Kenneth L. McNally
IRRI, Philippines

Comments from the editor:

Please register Ricepilaf in the bio.tools and SciCrunch.org databases to receive RRID (Research Resource Identification Initiative ID) and biotoolsID identifiers, and include these in your manuscript. Computational workflows should be registered in workflowhub.eu and the DOIs cited in the relevant places in the manuscript. These will facilitate tracking, reproducibility and re-use of your tool.

Response:
We have registered RicePilaf in the bio.tools and SciCrunch.org databases. The bio.tools URL and SciCrunch RRID have been added to the Availability of source code and requirements section of the manuscript.

Comments from Reviewer 1:

1) I suggest that gene expression information databases such as RiceXPro (https://ricexpro.dna.affrc.go.jp/) and Transcriptome ENcyclopedia Of Rice (https://tenor.dna.affrc.go.jp/) should also be made available. By referring to organ-specific expression information of genes, it could be possible to narrow down the causative genes.

We agree with the reviewer that having organ/tissue-specific gene expression information would help narrow down the candidate gene list from GWAS on traits that are organ/tissue-specific. We had discussed this issue in the Limitations section. We have expanded the discussion to include the RiceXPro and TENOR databases.

However we were not able to easily incorporate the two databases into our app. RiceXPro uses Agilent Microarray, and the NCBI GEO dataset does not provide a mapping of the microarray spot to RAP or RGAP7 IDs, making it not possible to integrate the results in NCBI GEO used in RiceXPro to the RicePilaf. On the other hand, the data available for download from the TENOR dataset contains only expression data from two organs: root and shoot and not comprehensive enough to build a candidate gene filter in RicePilaf. While in theory, we could utilize the TENOR dataset to instead build a co-expression network, we already employ two similarly built co-expression networks (RiceNet v2 and RCRN) in the app.

2) This paper presents the results of the analysis of two cases using RicePilaf. However, the paper only describes the results and discussion of each analysis section, and lacks the process of integrating these results and narrowing them down to the candidate genes. Given that the goal of this paper is to obtain

functional insights by multiplying multiple analysis sections, the route to this goal should be more clearly presented.

As a response to this comment, we have implemented a major feature update to integrate the results and to allow users to narrow down candidate genes.

The summary of results of the various analyses performed by the user is integrated into an interactive table and displayed in the Summary page. Rows of the table correspond to the candidate genes and columns contain summary statistics such as the number of pathways that contain the gene, the number of pathways/ontology terms that are associated with co-expression network cluster the gene belongs to, the number of articles the gene was found in our text-mining results, etc. The rows of the table can be sorted based on multiple columns, allowing the user to prioritize the genes based on criteria they deem important.

Comments from Reviewer 2:

1) As far as I know, there are already a lot of databases on rice genomes, transcriptomes, and epigenomics. What are the advantages of this platform? Please provide a specific picture statistic for those data included, refer to Figure 1A of https://doi.org/10.1093/nar/gkad701

RicePilaf is not a database, rather a platform to integrate information from various existing databases and provide tools that are lacking in other databases. One of the strengths of the app is that the entry point to it is a GWAS/QTL region, something other platforms do not directly address.

As suggested, we have added a figure (Figure 1 in the manuscript), which provides a graphical abstract of the databases used in the app. Similar information is also available in Table 4, which provides a summary of the databases used.

2) Please add some citations of existing rice star databases and compare the advantages of this data platform with these existing databases.

The databases that the tool uses are summarized in Table 4. Citations and URLs are provided in the table.

To reiterate, RicePilaf is not a database, rather a platform to integrate information from various existing databases. One of the strengths of the app is that the entry point to it is a GWAS/QTL region, something other platforms do not directly address.

3) The results of some existing quantitative traits are lacking, such as anaerobic germination (10.1002/fes3.354) and morphological traits (10.1111/tpj.15705 and 10.3389/fpls.2022.995634)

We have added the three citations.

4) In the abstract, "not all of which are causal, and many of which are in non-coding regions." Non-coding region variants are not necessarily not causal mutations, and it is suggested that this should be changed to "there are some false-positive results".

The sentence does not imply that non-coding region variants are not causal. Hence, we have not edited the statement.
