# Supplementary material for: RicePilaf: a post-GWAS/QTL dashboard to integrate pangenomic, coexpression, regulatory, epigenomic, ontology, pathway, and text-mining information to provide functional insights into rice QTLs and GWAS loci
Source: Gigascience. 2024 Jun 4;13:giae013. doi: 10.1093/gigascience/giae013 (PMC11148593; doi:10.1093/gigascience/giae013)
Supplement: giae013_Reviewer_1_Report_Original_Submission — Mao Suganami -- 11/20/2023 Reviewed [file giae013_reviewer_1_report_original_submission.pdf]

Reviewer Report

Title: RicePilaf: a post-GWAS/QTL dashboard to integrate pangenomic, co-expression, regulatory, epigenomic, ontology, pathway, and text-mining information to provide functional insights into rice QTLs and GWAS loci

Version: Original Submission **Date:** 11/20/2023

Reviewer name: Mao Suganami

Reviewer Comments to Author:

In this study, the authors developed RicePilaf as a useful tool for finding causal polymorphisms in rice GWAS/QTL studies. The system is designed to aggregate information necessary to search for causal polymorphisms, such as gene list output using multiple reference genomes, co-expression information, and related literature information by text mining, across publicly available databases. It is designed to be used on a web browser, and I believe it will be a useful tool for many researchers. To improve the system and the paper, we request that the authors consider revising the following two points.1. Utilization of databases of gene expression informationI suggest that gene expression information databases such as RiceXPro (https://ricexpro.dna.affrc.go.jp/) and Transcriptome ENcyclopedia Of Rice (https://tenor.dna.affrc.go.jp/) should also be made available. By referring to organ-specific expression information of genes, it could be possible to narrow down the causative genes.2. Demonstration of use caseThis paper presents the results of the analysis of two cases using RicePilaf. However, the paper only describes the results and discussion of each analysis section, and lacks the process of integrating these results and narrowing them down to the candidate genes. Given that the goal of this paper is to obtain functional insights by multiplying multiple analysis sections, the route to this goal should be more clearly presented.I believe that a very useful tool has been developed, and hope that the above two points will be addressed and brushed up the paper.

Level of Interest

Please indicate how interesting you found the manuscript: Choose an item.

Quality of Written English

Please indicate the quality of language in the manuscript: Choose an item.

Declaration of Competing Interests

Please complete a declaration of competing interests, considering the following questions:

- Have you in the past five years received reimbursements, fees, funding, or salary from an organisation that may in any way gain or lose financially from the publication of this manuscript, either now or in the future?

- Do you hold any stocks or shares in an organisation that may in any way gain or lose financially from the publication of this manuscript, either now or in the future?
- Do you hold or are you currently applying for any patents relating to the content of the manuscript?
- Have you received reimbursements, fees, funding, or salary from an organization that holds or has applied for patents relating to the content of the manuscript?
- Do you have any other financial competing interests?
- Do you have any non-financial competing interests in relation to this paper?

If you can answer no to all of the above, write 'I declare that I have no competing interests' below. If your reply is yes to any, please give details below.

I declare that I have no competing interests

I agree to the open peer review policy of the journal. I understand that my name will be included on my report to the authors and, if the manuscript is accepted for publication, my named report including any attachments I upload will be posted on the website along with the authors' responses. I agree for my report to be made available under an Open Access Creative Commons CC-BY license (http://creativecommons.org/licenses/by/4.0/). I understand that any comments which I do not wish to be included in my named report can be included as confidential comments to the editors, which will not be published.

Choose an item.

To further support our reviewers, we have joined with Publons, where you can gain additional credit to further highlight your hard work (see: https://publons.com/journal/530/gigascience). On publication of this paper, your review will be automatically added to Publons, you can then choose whether or not to claim your Publons credit. I understand this statement.

Yes Choose an item.
