# Supplementary material for: RicePilaf: a post-GWAS/QTL dashboard to integrate pangenomic, coexpression, regulatory, epigenomic, ontology, pathway, and text-mining information to provide functional insights into rice QTLs and GWAS loci
Source: Gigascience. 2024 Jun 4;13:giae013. doi: 10.1093/gigascience/giae013 (PMC11148593; doi:10.1093/gigascience/giae013)
Supplement: giae013_Reviewer_2_Report_Original_Submission — Weilong Kong -- 11/23/2023 Reviewed [file giae013_reviewer_2_report_original_submission.pdf]

Reviewer Report

Title: RicePilaf: a post-GWAS/QTL dashboard to integrate pangenomic, co-expression, regulatory, epigenomic, ontology, pathway, and text-mining information to provide functional insights into rice QTLs and GWAS loci

Version: Original Submission **Date:** 11/23/2023

Reviewer name: Weilong Kong

Reviewer Comments to Author:

Anish et al., developed a platform (RicePilaf) to integratepangenomic, co-expression, regulatory, epigenomic, ontology, pathway, and text-mining information toontology, pathway, and text-mining information toprovide functional insights into rice QTLs and GWAS loci. This is of practical significance for the analysis of complex biological mechanisms in rice, and enables the integration of multi-omics data to target causal genes without switching platforms.There are only a few minor issues I need to address here.1. As far as I know, there are already a lot of databases on rice genomes, transcriptomes, and epigenomics. What are the advantages of this platform? Please provide a specific picture statistic for those data included, refer to Figure 1A of https://doi.org/10.1093/nar/gkad701.2. Please add some citations of existing rice star databases and compare the advantages of this data platform with these existing databases.3. The results of some existing quantitative traits are lacking, such as anaer-obic germination (10.1002/fes3.354) and morphological traits (10.1111/tpj.15705 and 10.3389/fpls.2022.995634)4. In the abstract, "not all of which are causal, and many ofIn the abstract, "not all of which are causal, and many of which are in non-coding regions." Non-coding region variants are not necessarily not causal mutations, and it is suggested that this should be changed to "there are some false-positive results".

Level of Interest

Please indicate how interesting you found the manuscript: Choose an item.

Quality of Written English

Please indicate the quality of language in the manuscript: Choose an item.

Declaration of Competing Interests

Please complete a declaration of competing interests, considering the following questions:

- Have you in the past five years received reimbursements, fees, funding, or salary from an organisation that may in any way gain or lose financially from the publication of this manuscript, either now or in the future?

- Do you hold any stocks or shares in an organisation that may in any way gain or lose financially from the publication of this manuscript, either now or in the future?
- Do you hold or are you currently applying for any patents relating to the content of the manuscript?
- Have you received reimbursements, fees, funding, or salary from an organization that holds or has applied for patents relating to the content of the manuscript?
- Do you have any other financial competing interests?
- Do you have any non-financial competing interests in relation to this paper?

If you can answer no to all of the above, write 'I declare that I have no competing interests' below. If your reply is yes to any, please give details below.

I have no competing interests' below

I agree to the open peer review policy of the journal. I understand that my name will be included on my report to the authors and, if the manuscript is accepted for publication, my named report including any attachments I upload will be posted on the website along with the authors' responses. I agree for my report to be made available under an Open Access Creative Commons CC-BY license (http://creativecommons.org/licenses/by/4.0/). I understand that any comments which I do not wish to be included in my named report can be included as confidential comments to the editors, which will not be published.

Choose an item.

To further support our reviewers, we have joined with Publons, where you can gain additional credit to further highlight your hard work (see: https://publons.com/journal/530/gigascience). On publication of this paper, your review will be automatically added to Publons, you can then choose whether or not to claim your Publons credit. I understand this statement.

Yes Choose an item.
